# Interplay of SHH, WNT and BMP4 signaling regulates the development of the lamina propria in the murine ureter

**DOI:** 10.1242/dev.204214

**Published:** 2025-02-06

**Authors:** Philipp Straube, Anja Beckers, Ulrich W. H. Jany, Florian Bergmann, Timo H.-W. Lüdtke, Carsten Rudat, Mark-Oliver Trowe, Imke Peters, Maximilian G. Klopf, Tamrat M. Mamo, Andreas Kispert

**Affiliations:** Institute of Molecular Biology, Hannover Medical School, 30625 Hannover, Germany

**Keywords:** Ureter, Lamina propria, Differentiation, Organogenesis, Signaling

## Abstract

In mammalian ureters, the lamina propria presents as a prominent layer of connective tissue underneath the urothelium. Despite its important structural and signaling functions, little is known how the lamina propria develops. Here, we show that in the murine ureter the lamina propria arises at late fetal stages and massively increases by fibrocyte proliferation and collagen deposition after birth. WNT, SHH, BMP4 and retinoic acid signaling are all active in the common mesenchymal progenitor of smooth muscle cells and lamina propria fibrocytes. However, around birth, the lamina propria becomes a target for epithelial WNT and SHH signals and a source of BMP4 and retinoic acid. SHH and WNT signaling promote lamina propria and smooth muscle cell differentiation and proliferation at fetal and early postnatal stages, whereas BMP4 signaling is required for early smooth muscle cell differentiation but not for its later maintenance. Our findings suggest that, in the presence of SHH and WNT signaling, it is the modulation of BMP4 signaling which is the major determinant for the segregation of lamina propria and smooth muscle cells.

## Teaser

**Summary:** Epithelial SHH and WNTs are crucial signals for the proliferation and differentiation of the lamina propria in the fetal and early postnatal murine ureter. Absence of BMP4 signaling segregates the lamina propria from the adjacent smooth muscle cell layer.

## INTRODUCTION

The lamina propria (LP) is a loose connective tissue that underlies the inner epithelial lining of most tubular organs. It provides mechanical support by tethering the epithelium to the surrounding mesenchymal wall. Moreover, its abundant and flexible extracellular matrix serves as a bed for various types of immune cells, capillary endothelial cells and nerve endings to fight external pathogens, nurture the epithelium, and sense and respond to stimuli from the luminal content (Andersson and McCloskey, 2014; Roulis and Flavell, 2016).

In the main organs of the urinary drainage system, the ureter and the bladder, the LP is highly compressible and elastic due to a meshwork of thick interwoven and crisscrossing collagen fibers that, upon dilatation, change their orientation to lie parallel to the specialized pseudo-stratified epithelium of the urinary drainage system, the urothelium (Aitken and Bägli, 2009; Macarak and Howard, 1997). The LP does not only play a structural role, it has been recognized as a crucial source of signals for the growth, differentiation and maintenance of the urothelium and the adjacent tunica muscularis (TM) (Andersson and McCloskey, 2014; Chun et al., 2007; Gandhi et al., 2013; Mysorekar et al., 2009).

Despite these important functions, the cellular and molecular mechanisms that control the development and homeostasis of the LP in the excretory system as in other tubular organs, have remained poorly understood. Genetic lineage tracing revealed that in the developing ureter of the mouse, LP fibrocytes arise together with smooth muscle cells (SMCs) of the TM and fibrocytes of the outer tunica adventitia (TA) from a common mesenchymal progenitor pool that surrounds the epithelium of the distal ureteric bud (Bohnenpoll et al., 2013) at embryonic day (E) 11.5. At E12.5, mesenchymal cells adjacent to the ureteric epithelium (UE) acquire a rhomboid shape and start to express the target of canonical WNT signaling *Axin2*. These *Axin2^+^* progenitors activate at E14.5 the SMC regulator *Myocd* (Trowe et al., 2012; Wang and Olson, 2004). Cells in the direct vicinity of the UE switch *Myocd* expression off and differentiate into fibrocytes of the LP. The ones further away maintain *Myocd* expression and activate, in a stepwise fashion, expression of different SMC structural genes. *Axin2^−^* cells in the outer region of the ureteric mesenchyme (UM) are lineage segregated from E13.5 onwards and differentiate into adventitial fibrocytes (Bohnenpoll et al., 2017a).

Tissue separation and recombination experiments have shown that development of the early (undifferentiated) UM depends on signals from the UE (Bohnenpoll and Kispert, 2014; Cunha, 1976). Subsequent work primarily focused on the signaling pathways and their transcriptional output that drive the differentiation of SMCs from the mesenchymal progenitor pool (Bohnenpoll and Kispert, 2014). SHH and WNTs (most likely WNT7B) were characterized as epithelial signals important for the proliferation of the mesenchymal progenitors and the differentiation of SMCs (Bohnenpoll et al., 2017c; Trowe et al., 2012; Yu et al., 2002). Downstream or parallel to these epithelial signals, mesenchymal BMP4 is required for mesenchymal expansion and SMC differentiation, whereas retinoic acid (RA) from both the UE and the UM maintains the undifferentiated character of the UM (Bohnenpoll et al., 2017b; Mamo et al., 2017; Wang et al., 2009). Interestingly, increased SHH signaling was associated with expansion of the LP in the ureter and bladder (Ikeda et al., 2017; Yu et al., 2002).

Here, we used time-resolved pharmacological inhibition and activation experiments in explant cultures to decipher the signaling inputs for the development of the LP in the murine ureter. We provide evidence that in the presence of SHH and WNT signaling, it is the lack of BMP4 signaling that favors the LP over the SMC fate.

## RESULTS

### The LP expands largely after birth

**Fig. 1.**
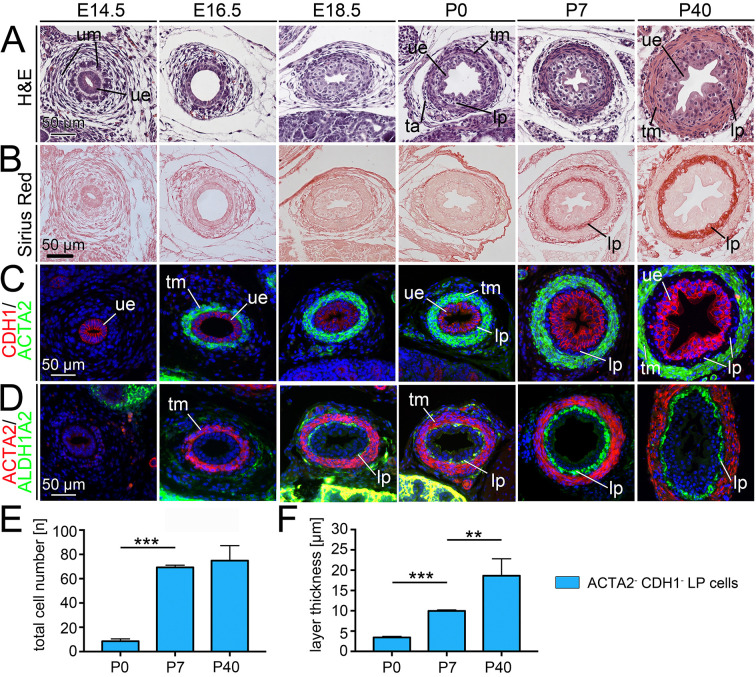
**The development of the lamina propria in the murine ureter.** (A-D) Histological analysis by Hematoxylin and Eosin (H&E) (A) and Picrosirius Red (Sirius Red) stainings (B), and co-immunofluorescence analysis of expression of the smooth muscle cell marker ACTA2 and the ureteric epithelium marker CDH1 (C), and of the smooth muscle cell marker ACTA2 and the lamina propria marker ALDH1A2 (D) on transverse sections of the proximal ureter in wild-type mice from E14.5 to P40. Nuclei in immunofluorescence images are counter-stained with DAPI (blue). *n*=5 for each assay and each stage. (E) Quantification of LP fibrocytes by counting of DAPI^+^ nuclei in the layer of ACTA2^−^CDH1^−^ suburothelial mesenchymal cells on transverse sections of the proximal ureter region at P0, P7 and P40. (F) Quantification of the thickness of the LP layer on transverse sections of the proximal ureter region at P0, P7 and P40. Five distinct positions per section were measured and averaged. *n*=5 for each assay. For numbers and statistics (two-tailed Student's *t*-test, Welch's *t*-test or Mann–Whitney *U*-test) see Table S1. *P<0.05; **P<0.01; ***P<0.001. Data are mean±s.d. c, control; lp, lamina propria; ta, tunica adventitia; tm, tunica muscularis; ue, ureteric epithelium; um, ureteric mesenchyme.

To characterize LP development in the murine ureter, we performed both histological stainings as well as immunofluorescence analysis of marker proteins on transverse sections of the proximal ureter region in wild-type mice at prenatal and postnatal stages. Hematoxylin and Eosin staining discriminated cell layers due to the differential distribution of acid (Hematoxylin) and basic structures (Eosin) (Fischer et al., 2008) (Fig. 1A). Sirius Red visualized the deposition of collagens in the extracellular matrix (Sweat et al., 1964) (Fig. 1B). CDH1 marked the basolateral membranes of the UE. Combinatorial cytoplasmic expression of ACTA2 and TAGLN identified SMCs of an early differentiation stage; a late stage of differentiation was characterized by additional expression of CKM and SMTN in the cytoplasm of these cells (Bohnenpoll et al., 2017a; Kurz et al., 2022b) (Fig. 1C; Fig. S1). Based on findings that *Aldh1a2* expression marks the LP at E18.5 (Bohnenpoll et al., 2017b; Trowe et al., 2012), we used ALDH1A2 as a marker for the cytoplasm of LP fibrocytes (Deuper et al., 2022) (Fig. 1D). COL1A2, CDH2, DES, THY1 and VIM, which have been used as LP markers in other biological contexts (Kuijpers et al., 2014; Poletajew et al., 2015; Powell et al., 2011; Roulis and Flavell, 2016), proved unsuitable in the ureter (Fig. S2).

At the end of the undifferentiated phase, at E14.5, the UM appeared to be histologically subdivided into a dense inner region of large cuboidal cells and an outer region of loosely organized fibrocytic cells. The UE was positive for CDH1; the UM neither expressed SMC markers nor ALDH1A2 at this stage. At E16.5, the cells of the inner region of the UM co-expressed ACTA2 and TAGLN, indicating early SMC differentiation. At E18.5 and postnatal day (P) 0, patches of ALDH1A2^+^ cells were found in between the ACTA2^+^TAGLN^+^CKM^+^SMTN^+^ late SMCs and the CDH1^+^ urothelium. At P7 and P40, the population of SMC marker-negative mesenchymal cells underneath the urothelium appeared to be largely expanded, and expressed ALDH1A2 homogenously. Sirius Red staining indicated a massive deposition of collagens in the LP (Fig. 1; Fig. S1). Quantification of ACTA2^−^CDH1^−^ suburothelial mesenchymal cells at postnatal stages confirmed a strong increase of LP fibrocytes between P0 and P7 (Fig. 1E; Table S1A). The LP layer gained some thickness in this time interval but further expanded until P40 (Fig. 1F; Table S1B). We conclude that ALDH1A2 expression allows robust detection of LP fibrocytes from E18.5/P0 until at least P40. The major expansion of the LP occurs after birth and is associated with a strong increase of LP fibrocytes until P7, and collagen deposition until and after this stage.

### The LP is a source and a target of signals

**Fig. 2.**
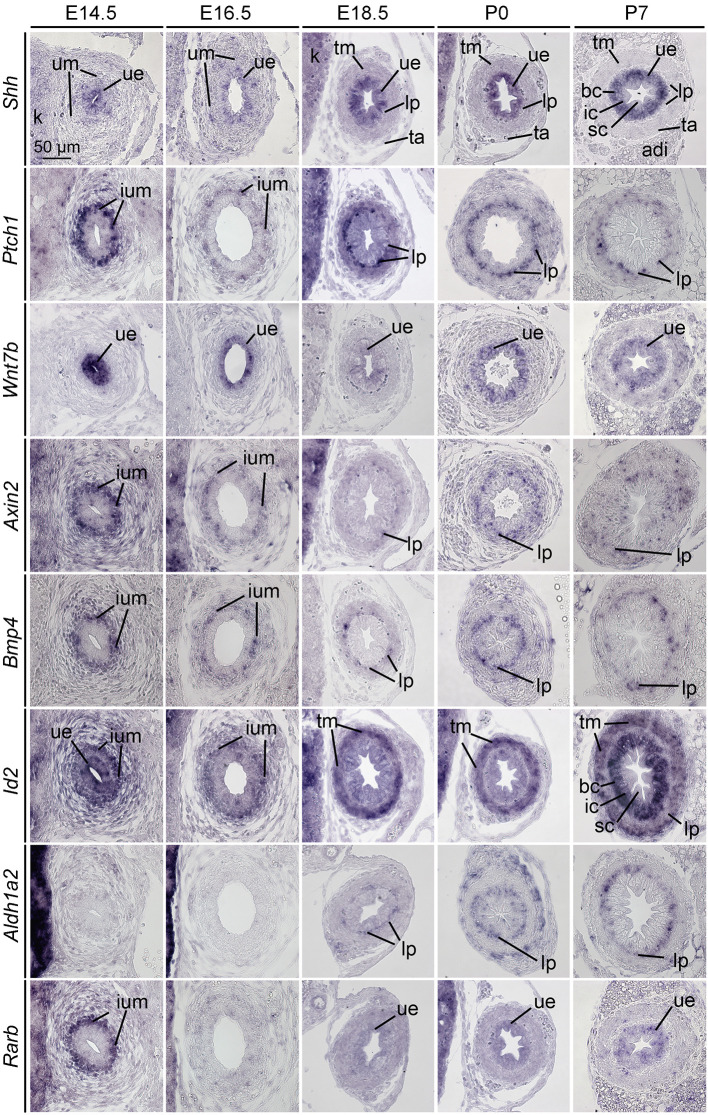
**Activity of signaling pathways during lamina propria development.** RNA *in situ* hybridization analysis of expression of *Shh* and the SHH signaling target gene *Ptch1*; of *Wnt7b* and the WNT signaling target gene *Axin2*; of *Bmp4* and the BMP signaling target gene *Id2*; of the gene encoding the retinoic acid synthesizing enzyme *Aldh1a2*, and the target gene of retinoic acid signaling *Rarb*, on transverse sections of the proximal ureter of wild-type embryos at prenatal (E14.5, E16.5, E18.5) and postnatal (P0, P7) stages. *n*≥5 for all probes and stages. adi, adipose tissue; bc, B-cells; ic, I-cell; ium, inner region of the ureteric mesenchyme; k, kidney; lp, lamina propria; sc, S-cell; ta, tunica adventitia; tm, tunica muscularis; ue, ureteric epithelium; um, ureteric mesenchyme.

Previous work has implicated SHH, WNT, BMP4 and RA signaling in the development of ureteric SMCs (Bohnenpoll et al., 2017b,c; Mamo et al., 2017; Trowe et al., 2012; Yu et al., 2002). To investigate whether these pathways are also associated with LP development, we performed section RNA *in situ* hybridization analysis to profile the expression of genes encoding the signals as well as targets of their activity during ureter development (Fig. 2).

*Shh* was expressed at low levels in the UE at E14.5 and E16.5. From E18.5 onwards, increased expression was confined to the basal and intermediate cell layer of the UE. Expression of *Ptch1*, encoding a receptor for SHH and presenting a target gene of SHH signaling (Ingham and McMahon, 2001), was found in the inner region of the UM at E14.5 and E16.5, and from E18.5 onwards in the LP. *Wnt7b* was strongly expressed in the UE at E14.5 and E16.5; at subsequent stages expression was found at lower levels in the urothelium, most likely in B- and I-cells. Expression of *Axin2*, a direct target gene of WNT signaling (Jho et al., 2002), mirrored the spatial pattern of *Ptch1*. Expression of *Bmp4* was found in the inner UM at E14.5 and E16.5. From E18.5 onwards, expression was restricted to the LP. Expression of *Id2*, a direct target gene of BMP signaling (Hollnagel et al., 1999), occurred at high levels both in the UE and the UM at E14.5. Expression continued at E16.5 in the inner region of the UM, and at E18.5 to P7 in the TM. At P7, expression also occurred in the basal and intermediate layers of the UE. The gene encoding the RA-synthesizing enzyme *Aldh1a2* was expressed in the LP at E18.5, P0 and P7. *Rarb*, encoding a receptor of RA and presenting a direct target gene of RA signaling (Mendelsohn et al., 1991), was expressed in the inner UM at E14.5. From E18.5 to P7, weak expression was found in the UE. We conclude that SHH, WNT, BMP4 and RA signaling occur strongly at E14.5 in the inner region of the undifferentiated UM where the common progenitor of SMCs and LP fibrocytes resides. From E18.5 onwards, the LP becomes a target of SHH and WNT and a source of BMP4 and RA signals.

### Pharmacological approaches allow stage-specific inhibition and activation of SHH, WNT, BMP4 and RA signaling in ureteral explant cultures

To characterize the individual and combinatorial function of these signaling pathways in LP development, we wished to employ a pharmacological approach in ureteral explant cultures. Centered on concentrations that are known to affect ureteric SMC development (Bohnenpoll et al., 2017b,c; Trowe et al., 2012), we first tested the efficacy of pathway inhibitors and activators for two developmental stages. For this, we explanted ureters together with kidneys at E12.5 (please note that embryonic ureters grow better in this combination culture and stick better on the membrane during later transfer steps) and ureters only at P0 on polyester membranes, cultured them for 18 h at the air-liquid interface in a range of concentrations of the compounds, and performed whole-mount RNA *in situ* hybridization for direct target genes as a read-out (see above; Figs S3 and S4). For SHH signaling (read-out: *Ptch1*), we found robust pathway inhibition by the SMO antagonist cyclopamine at 5 and 10 µM, and activation by the SMO agonist purmorphamine at 2 and 10 µM (Bohnenpoll et al., 2017c; Chen et al., 2002; Cooper et al., 1998). The WNT pathway (read-out: *Axin2*) was inhibited by the Porcupine inhibitor IWP-2 at 5 and 10 µM and activated by the GSK-3 inhibitor BIO at 10 and 20 µM (Karner et al., 2011; Sato et al., 2004). The BMP4 pathway (read-out: *Id2*) was inhibited by recombinant Noggin (NOG) at 5 and 10 µg/ml, and activated by recombinant BMP4 at 100 and 250 ng/ml (Bohnenpoll et al., 2017c; Mamo et al., 2017; Zimmerman et al., 1996). Inhibition of RA signaling (read-out: *Rarb*) was achieved by the pan-RAR antagonist BMS493 at 1 and 5 µM. Overactivation occurred with RA at the same concentrations (Bohnenpoll et al., 2017b; Chazaud et al., 2003). Hence, robust pathway inhibition and activation can be achieved for SHH, WNT, BMP4 and RA signaling in ureteral explant cultures at fetal and early postnatal stages.

### SHH and WNT signaling are required for fetal LP development

**Fig. 3.**
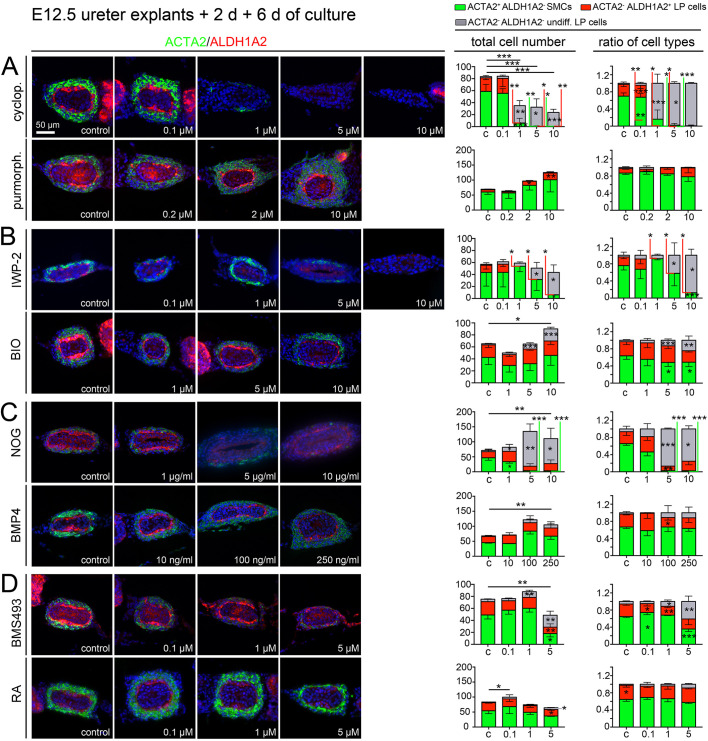
**Pharmacological analysis of signaling pathways in the early (fetal) development of the lamina propria.** (A-D) Embryonic ureters and kidneys were explanted at E12.5 and cultured for 2 days before they were incubated for another 6 days with increasing concentrations of pharmacological inhibitors or activators of SHH signaling [0.1-10 µM cyclopamine (cyclop.), 0.2-10 µM purmorphamine (purmorph.)] (A), WNT signaling (0.1-10 µM IWP-2, 1-10 µM BIO) (B), BMP4 signaling (1-10 µg/ml NOG, 10-250 ng/ml BMP4) (C), and retinoic acid (RA) signaling (0.1-5 µM BMS493, 0.1-5 µM RA) (D). Cultures were then processed for expression of the smooth muscle cell (SMC) marker ACTA2 and the lamina propria (LP) marker ALDH1A2 (left panel), and the total number of cells and the ratio of ACTA2^+^ALDH1A2^−^ SMCs (green), ACTA2^−^ALDH1A2^+^ LP fibrocytes (red), and ACTA2^−^ALDH1A2^−^ ‘undifferentiated’ mesenchymal cells (gray) were determined on proximal ureter sections (right panel). *n*=5 for each assay. For numbers and statistics (two-tailed Student's *t*-test, Welch's *t*-test or Mann–Whitney *U*-test) see Tables S2A-S9A. *P<0.05; **P<0.01; ***P<0.001. Data are mean±s.d. c, control.

To analyze the role of these signaling pathways in the fetal period of LP development (when LP fibrocytes arise together with SMCs from a common mesenchymal progenitor), we explanted E12.5 wild-type ureters (with kidneys) and cultured them for 2 days without drug to reach a stage similar to E14.0 to E14.5 ureters *in vivo* (Fig. S5), before we added inhibitors and activators in the same concentration range as above for 6 days (Figs S3 and S4). We performed immunofluorescence analysis on sections for expression of the SMC marker ACTA2 and the LP marker ALDH1A2 (Fig. 3, left panel) and quantified the number and ratio of ACTA2^+^ALDH1A2^−^ SMCs, ACTA2^−^ALDH1A2^+^ differentiated LP fibrocytes and ACTA2^−^ALDH1A2^−^ ‘undifferentiated’ mesenchymal cells (Fig. 3, right panel; Tables S2A-S9A).

Treatment with cyclopamine led to a strong reduction of mesenchymal cells and an almost complete loss of differentiated ACTA2^+^ SMCs and ALDH1A2^+^ LP fibrocytes from 1 µM onwards (Fig. 3A, first row; Table S2A). Treatment with purmorphamine led to a dose-dependent increase of both differentiated SMCs and LP fibrocytes (Fig. 3A, second row; Table S3A). Inhibition of WNT signaling by IWP-2 resulted in a reduction of mesenchymal cells, with a loss of differentiated LP fibrocytes and a dose-dependent reduction of SMCs from 1 to 10 µM of the inhibitor (Fig. 3B, first row; Table S4A). BIO treatment led to a dose-dependent increase of the overall mesenchymal cell number largely due to gain of ‘undifferentiated’ LP cells (Fig. 3B, second row; Table S5A). NOG treatment led to a dose-dependent loss of SMCs, accompanied by a gain of undifferentiated mesenchymal cells. The number of ALDH1A2^+^ cells remained largely unchanged (Fig. 3C, first row; Table S6A). Treatment with BMP4 increased the number of SMCs and of undifferentiated LP cells (at 100 and 250 ng/ml) (Fig. 3C, second row; Table S7A). Inhibition of RA signaling did not affect mesenchymal differentiation and cell number in a clear dose-dependent way. However, at 5 µM BMS493 treatment, SMCs and differentiated LP cells were strongly reduced while undifferentiated mesenchymal cells were increased (Fig. 3D, first row; Table S8A). Treatment with RA led to a small reduction of LP cells at the highest dose of 5 µM (Fig. 3D, second row; Table S9A).

To validate that changes of ALDH1A2 expression reflect changes in the number of LP fibrocytes (and not merely the loss or gain of a marker), we additionally analyzed ACTA2 and CDH1 expression, identifying LP fibrocytes as ACTA2^−^CDH1^−^ (unstained) cells in between the TM and the UE (Fig. S6, left panel). The changes of numbers and ratios of LP cells and SMCs were congruent with the above findings (Fig. S6, right panel; Tables S2B-S9B). Together with the fact that the dose response curves fit to the findings of pathway inhibition and activation (Figs S3 and S4), we conclude that in fetal ureter development SHH and WNT signaling are positive regulators of differentiation and expansion of LP fibrocytes and SMCs from the common progenitor. BMP4 signaling is required for differentiation of SMCs but not for LP cells.

### SHH and WNT signaling are required for postnatal LP development

**Fig. 4.**
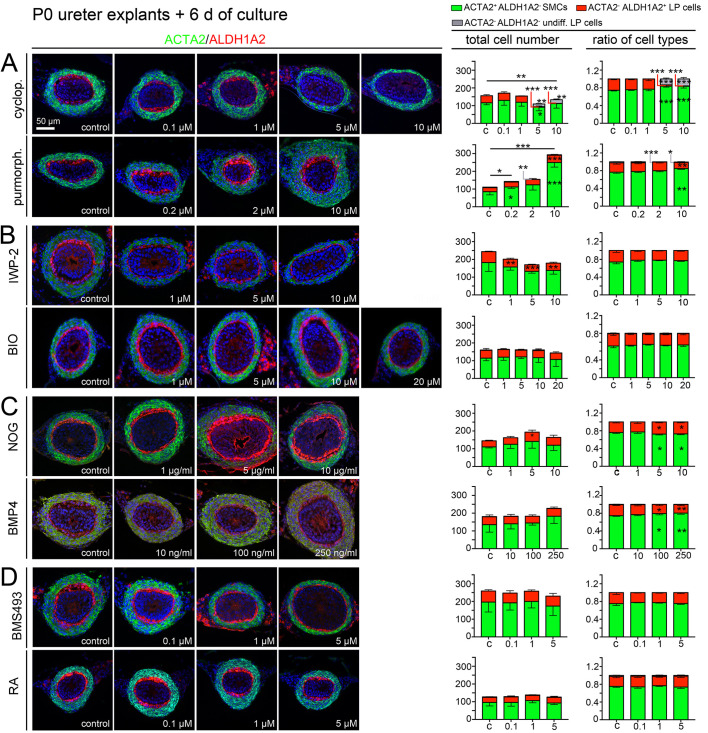
**Pharmacological analysis of signaling pathways in the postnatal development of the lamina propria.** (A-D) Embryonic ureters were explanted at P0 and cultured for 6 days in the presence of increasing concentrations of pharmacological inhibitors or activators of SHH signaling [0.1-10 µM cyclopamine (cyclop.), 0.2-10 µM purmorphamine (purmorph.)] (A), WNT signaling (1-10 µM IWP-2, 1-20 µM BIO) (B), BMP4 signaling (1-10 µg/ml NOG, 10-250 ng/ml BMP4) (C), and retinoic acid (RA) signaling (0.1-5 µM BMS493, 0.1-5 µM retinoic acid) (D). Cultures were then processed for expression of the smooth muscle cell (SMC) marker ACTA2 and the lamina propria (LP) marker ALDH1A2 (left panel), and the total number of cells and the ratio of ACTA2^+^ALDH1A2^−^ SMCs (green), ACTA2^−^ALDH1A2^+^ LP fibrocytes (red), and ACTA2^−^ALDH1A2^−^ ‘undifferentiated’ mesenchymal cells (gray) were determined on proximal ureter sections (right panel). *n*=5 for each assay. For numbers and statistics (two-tailed Student's *t*-test, Welch's *t*-test or Mann–Whitney *U*-test) see Tables S10A-S17A. *P<0.05; **P<0.01; ***P<0.001. Data are mean±s.d. c, control.

We next investigated whether these signaling pathways also affect the early postnatal phase of LP development (when LP fibrocytes largely increase in number) by culturing P0 ureteral explants for 6 days with increasing concentrations of compounds as above. We again performed immunofluorescence analysis on sections for expression of the SMC marker ACTA2 and the LP marker ALDH1A2 (Fig. 4, left panel) and quantified the number and ratio of ACTA2^+^ALDH1A2^−^ SMCs, ACTA2^−^ALDH1A2^+^ differentiated LP fibrocytes and ACTA2^−^ALDH1A2^−^ ‘undifferentiated’ mesenchymal cells (Fig. 4, right panel; Tables S10A-S17A).

Inhibition of SHH signaling with 5 and 10 µM cyclopamine led to a loss of ALDH1A2 expression and a reduction of the mesenchymal cell number, with a stronger reduction of ‘LP cells’ than of SMCs (Fig. 4A, first row; Table S10A). Purmorphamine led to dose-dependent increase of both SMCs and LP fibrocytes; SMCs were disproportionally increased at 10 µM (Fig. 4A, second row; Table S11A). Inhibition of WNT signaling by IWP-2 led to a dose-dependent reduction of LP fibrocytes; the number of SMCs was weakly reduced (Fig. 4B, first row; Table S12A). BIO treatment was without effect (Fig. 4B, second row; Table S13A). Inhibition of BMP4 signaling led to a slight increase of ALDH1A2^+^ LP fibrocytes (peaking at 5 µg/ml NOG) (Fig. 4C, first row; Table S14A). BMP4 treatment increased the ratio of SMCs to LP fibrocytes at 100 and 250 ng/ml, probably due to a slight (but insignificant) increase of the total SMC number. In either case, the effect was weak and lacked a clear dose-response curve (Fig. 4C, second row; Table S15A). Manipulation of RA signaling did not affect the number of LP fibrocytes and SMCs at this stage (Fig. 4D; Tables S16A and S17A).

Analysis of ACTA2 and CDH1 expression, and quantification of SMCs as ACTA2^+^ cells and LP fibrocytes as ACTA2^−^CDH1^−^ (unstained) cells in between the TM and the UE were congruent with the above findings (Fig. S7; Tables S10B-S17B).

These findings suggest that SHH signaling is required for maintenance of ALDH1A2^+^ LP fibrocytes, whereas both SHH and WNT signaling account for the increase of the number of LP fibrocytes after birth. BMP4 signaling seems to slightly favor SMCs over LP fibrocytes, while RA signaling is irrelevant for postnatal LP development.

### SHH and WNT signaling cooperate in the postnatal expansion of LP cells

**Fig. 5.**
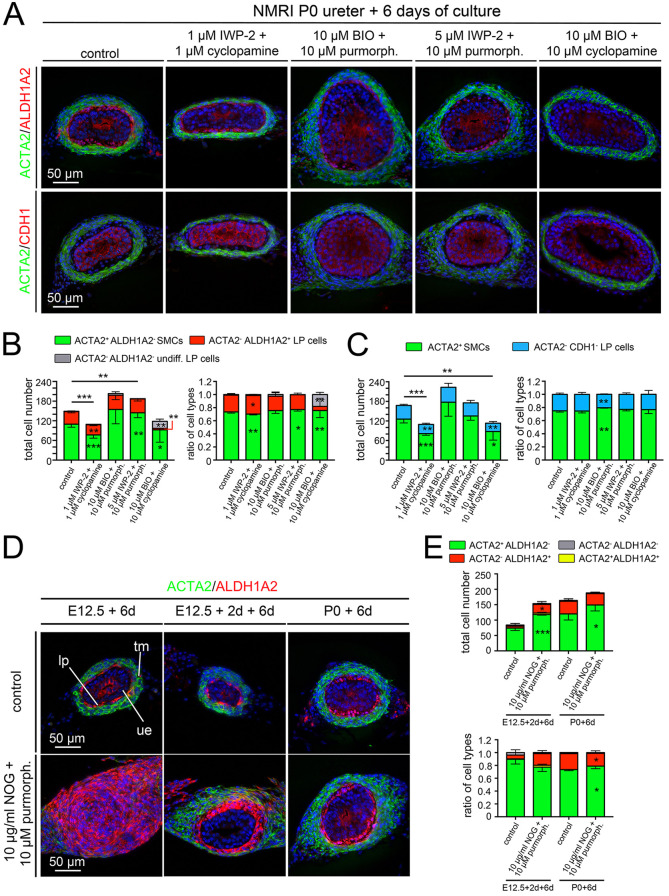
**Combinatorial pharmacological analysis of WNT/SHH and SHH/BMP4 signaling pathways in postnatal development of the lamina propria.** (A-C) Ureters were explanted at P0 and cultured for 6 days. The SHH signaling activator purmorphamine (10 µM, purmorph.) or the inhibitor cyclopamine (1 or 10 µM) were combined with either an activator (BIO, 10 µM) or inhibitor (IWP-2, 1 or 5 µM) of canonical WNT signaling. Cultures were then processed for co-immunofluorescence analysis of the smooth muscle cell (SMC) marker ACTA2 and the lamina propria (LP) marker ALDH1A2, and of ACTA2 and the urothelial marker CDH1 on proximal ureter sections (A). The total number of ACTA2^+^ALDH1A2^−^ SMCs (green), ACTA2^−^ALDH1A2^+^ LP fibrocytes (red) and ‘undifferentiated’ (undiff.) ACTA2^−^ALDH1A2^−^ LP cells (gray) as well as their ratio were determined (B). Ureter sections were also quantified for ACTA2^+^CDH1^−^ SMCs (green) and ACTA2^−^CDH1^−^ LP cells (blue) (C). *n*=5 for each assay. For numbers and statistics (two-tailed Student's *t*-test, Welch's *t*-test or Mann–Whitney *U*-test) see Table S18. *P<0.05; **P<0.01; ***P<0.001. (D,E) Embryonic ureters were explanted at E12.5 (without and with 2 days of preculture) and at P0, and cultured for 6 days in the presence of both an inhibitor of BMP4 signaling (10 µg/ml NOG) and an activator of SHH signaling (10 µM purmorph.). Cultures were then processed for immunofluorescence analysis of the SMC marker ACTA2 and the LP marker ALDH1A2 on proximal ureter sections (D), and the total number of cells and the ratio of ACTA2^+^ALDH1A2^−^ SMCs (green), ACTA2^−^ALDH1A2^+^ LP fibrocytes (red), ACTA2^−^ALDH1A2^−^ undifferentiated LP cells (gray) and ACTA2^+^ALDH1A2^+^ cells (yellow) were determined (E). *n*=4 for each assay. For numbers and statistics (two-tailed Student's *t*-test, Welch's *t*-test or Mann–Whitney *U*-test) see Table S19. *P<0.05; ***P<0.001. Data are mean±s.d. lp, lamina propria; tm, tunica muscularis; ue, ureteric epithelium.

As both SHH and WNT signaling affected LP expansion in postnatal ureter explants, we characterized their epistatic relationship by combinatorial administration of pathway inhibitors and activators in explant cultures of P0 ureters (Fig. 5A-C; Table S18). Combined treatment with 1 µM of cyclopamine and IWP-2, i.e. concentrations which did not affect LP and SMC development (cyclopamine, Fig. 4A; Tables S10 and S11) or led to a weak reduction of LP fibrocytes only (IWP-2, Fig. 4B; Tables S12 and S13) resulted in a strong reduction of ALDH1A2^+^ LP fibrocytes and of ACTA2^+^ SMCs, enhancing the effects of individual pathway inhibition. Combined treatment with purmorphamine and BIO increased the total cell number but did not affect the ratio of mesenchymal cell types (as did purmorphamine treatment alone). Activation of SHH signaling rescued LP cell number in conditions of WNT inhibition. WNT signaling activation did not rescue the number of ALDH1A2^+^ differentiated LP cells when SHH signaling was inhibited, but did expand the number of undifferentiated LP cells. Analysis of ACTA2/CDH1 co-stainings confirmed these findings. We conclude that in early postnatal ureter development, SHH signaling accounts for differentiation of LP cells, whereas SHH signaling cooperates with WNT signaling in expansion of LP cells and SMCs.

### BMP4 suppresses SHH-induced ALDH1A2 expression in uncommitted mesenchymal progenitors

To resolve the stage-dependent interaction of BMP4 and SHH signaling in mesenchymal differentiation, we explanted ureters at E12.5 and P0, and combined BMP4 signaling inhibition with SHH signaling activation. In E12.5 ureteral explants co-treated with purmorphamine (10 µM) and NOG (10 µg/ml), all mesenchymal cells exclusively expressed ALDH1A2 after 6 days of culture. In E12.5 explants precultured for 2 days to resemble E14.5 ureters, the number of ALDH1A2^+^ LP fibrocytes and ACTA2^+^ SMCs was increased; some mesenchymal cells co-expressed both markers after 6 days of compound treatment. In P0 explant cultures, SMCs were increased (Fig. 5D,E; Table S19). This suggests that BMP4 suppresses SHH-induced ALDH1A2 expression in uncommitted mesenchymal progenitors at E12.5. In committed progenitors (E12.5+2 days), and even more in differentiated SMCs (P0), BMP4 signaling does not appear to be required to maintain the SMC phenotype, nor does it have a major repressive effect on ALDH1A2 expression and LP fate.

### SHH and WNT signaling affect the proliferation of LP fibrocytes in postnatal ureters

**Fig. 6.**
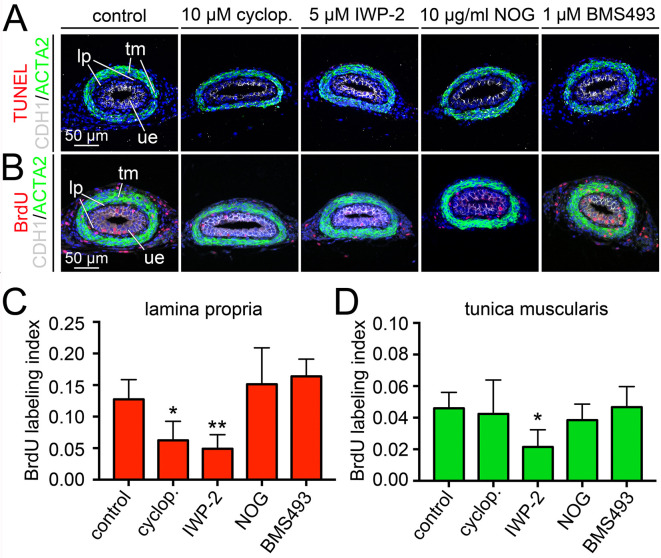
**Pharmacological analysis of signaling pathway dependency of apoptosis and proliferation in postnatal development of the lamina propria.** (A-D) Embryonic ureters were explanted at P0 and cultured for 18 h in the presence of inhibitors of signaling activities [10 µM cyclopamine (cyclop.), 5 µM IWP-2, 10 µg/ml NOG, 1 µM BMS493]. Cultures were then processed for immunofluorescence analysis of the smooth muscle cell marker ACTA2, the urothelial marker CDH1 and TUNEL (A), and for ACTA2, CDH1 and BrdU (B). The BrdU labeling index was determined for ACTA2^−^CDH1^−^ lamina propria fibrocytes (C) and ACTA2^+^CDH1^−^ smooth muscle cells (D). *n*≥4 for each assay. For numbers and statistics (two-tailed Student's *t*-test, Welch's *t*-test or Mann–Whitney *U*-test) see Table S20. *P<0.05; **P<0.01. Data are mean±s.d. lp, lamina propria; tm, tunica muscularis; ue, ureteric epithelium.

We next asked whether SHH, WNT, BMP4 and RA signaling affect apoptosis and/or proliferation in postnatal (P0) ureters. The TUNEL assay did not detect apoptotic bodies in P0 ureters cultured for 18 h with inhibitors of SHH, WNT, BMP4 or RA signaling (Fig. 6A). However, the BrdU incorporation assay revealed strongly reduced cell proliferation in the LP upon inhibition of SHH or WNT signaling. The latter also affected proliferation of SMCs. Inhibition of BMP4 and RA signaling did not affect mesenchymal cell proliferation (Fig. 6B-D; Table S20). This suggests that SHH and WNT signaling account for the proliferative expansion of the LP at postnatal stages.

### SHH and WNT signaling have common and distinct targets in the LP

**Fig. 7.**
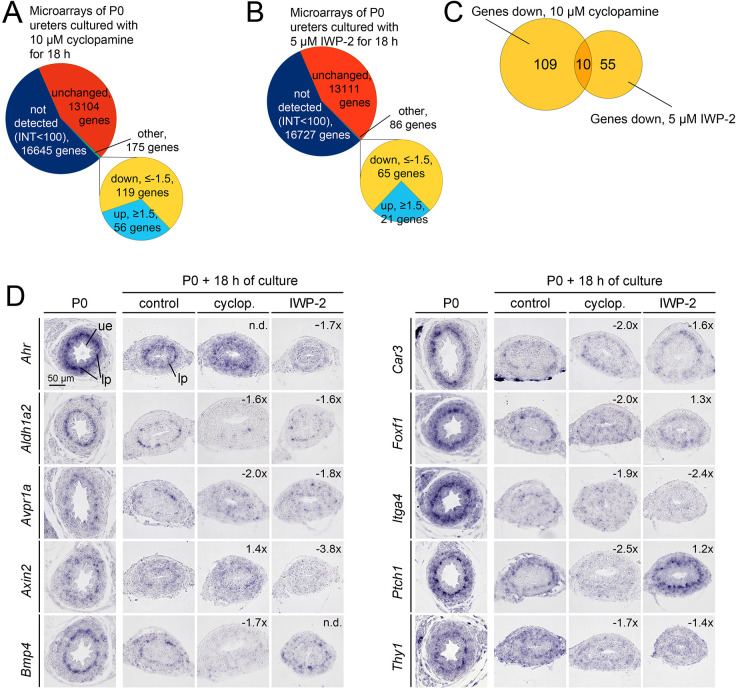
**Analysis of global transcriptional changes in P0 ureters treated for 18 h with inhibitors of SHH or WNT signaling.** (A,B) P0 ureters were explanted and treated for 18 h with 10 µM cyclopamine (A) or 5 µM IWP-2 (B) before transcriptional profiling was performed by microarray analysis and candidate gene expression was determined (Tables 1,2). Pie charts summarizing the results of the microarray analysis. (C) Venn diagram showing the intersection of the lists of genes with decreased expression in microarrays of 18 h cultures of cyclopamine- and IWP-2-treated P0 ureters. (D) RNA *in situ* hybridization analysis of expression of candidate genes on sections of the proximal ureter of P0 control embryos, and on P0 ureters cultured for 18 h in the presence of solvent (control), 10 µM cyclopamine (cyclop.) or 5 µM IWP-2. *n*=10 per probe and condition. Numbers in the upper right corner relate to the average fold change in the microarray. lp, lamina propria; ue, ureteric epithelium.

As SHH and WNT signaling act positively on both LP proliferation and/or differentiation, we explored whether they are interdependent and/or affect similar molecular programs. For this, we cultured P0 ureters for 18 h with the respective inhibitors (10 µM cyclopamine or 5 µM IWP-2), and profiled the transcriptional changes by microarray technology. Using filtering by intensity (>100 in control) and fold change (<−1.5), we found 119 genes with reduced expression in cyclopamine-treated ureters (Fig. 7A; Table S21; GSE282233). Functional annotation by DAVID revealed enrichment of terms related to forkhead transcription factors and various types of organ development in this gene set reflecting downregulation of *Foxl1*, *Foxf1* and *Foxd1* genes as well as of genes involved in various developmental programs including *Bmp4* and *Aldh1a2*. *Ptch1* expression was strongly reduced, validating SHH signaling inhibition (Tables 1A,2A; Table S22).

**Table 1.** Gene ontology (GO) enrichment analysis for downregulated genes following treatment with cyclopamine or IWP-2.

Rank	Term	*P*-value
**A Cyclopamine**		
1	GO:0005576∼extracellular region	6.3E-11
2	KW-0964∼Secreted	1.1E-07
3	GO:0005615∼extracellular space	1.9E-07
4	GO:0030324∼lung development	6.4E-07
5	mmu05200:Pathways in cancer	6.6E-06
6	GO:0005179∼hormone activity	7.9E-06
7	GO:0048566∼embryonic digestive tract development	3.4E-05
8	mmu04080:Neuroactive ligand-receptor interaction	4.7E-05
9	KW-0765∼Sulfation	1.1E-04
10	GO:0004957∼prostaglandin E receptor activity	1.2E-04
12	GO:0007507∼heart development	1.4E-04
13	GO:0021983∼pituitary gland development	2.0E-04
16	IPR018122:TF_fork_head_CS_1	3.1E-04
25	IPR030456:TF_fork_head_CS_2	9.1E-04
27	DNA_BIND:Fork-head	1.1E-03
29	IPR001766:Fork_head_dom	1.1E-03
**B IWP-2**		
1	CARBOHYF:N-linked (GlcNAc…) asparagine	1.0E-11
2	KW-0325∼Glycoprotein	1.7E-09
3	GO:0030178∼negative regulation of Wnt signaling pathway	1.7E-09
4	GO:OOO5576∼extracellular region	2.7E-08
5	KW-0879∼Wnt signaling pathway	5.4E-07
6	GO:0016055∼Wnt signaling pathway	5.8E-07
7	mmu04310:Wnt signaling pathway	1.9E-06
8	TOPO_DOM:Extracellular	2.9E-06
9	KW-0964∼Secreted	6.5E-06
10	GO:0042127∼regulation of cell population proliferation	4.2E-05

**Table 2.** Top 30 downregulated transcripts and additional candidates following treatment with cyclopamine or IWP-2.

**A Cyclopamine**			**B IWP-2**		
Rank	Gene	AvgFC	Rank	Gene	AvgFC
1	*Ibsp*	−28.1	1	*Cyp2e1*	−22.1
2	*Foxl1*	−6.4	2	*Ibsp*	−13.8
3	*Notumos*	−6.1	3	*Notumos*	−12.7
4	*Mc5r*	−4.7	4	*Lgr5*	−9.0
5	*Lrrc55*	−4.2	5	*Mc5r*	−7.9
6	*Ntng1*	−4.0	6	*Sp5*	−4.5
7	*Gli1*	−3.9	7	*Tmem132c*	−4.2
8	*Sct*	−3.9	8	*Tbx4*	−4.0
9	*Hhip*	−3.5	9	*Axin2*	−3.8
10	*Ripply1*	−3.2	10	*Ptger2*	−3.5
11	*Prss35*	−3.1	11	*Wipf3*	−2.8
12	*Ndp*	−3.1	12	*Gdpd2*	−2.7
13	*Rbp4*	−2.9	13	*Moxd1*	−2.7
14	*Rhox5*	−2.7	14	*Stra6*	−2.6
15	*Itih3*	−2.6	15	*Tcf7*	−2.6
16	*Sp5*	−2.6	16	*Wif1*	−2.6
17	*Fmod*	−2.6	17	*D630010B17Rik*	−2.5
18	*Ptch1*	−2.5	18	*Ptger3*	−2.4
19	*Ccl11*	−2.5	19	*Itga4*	−2.4
20	*Dio3*	−2.5	20	*Ptprr*	−2.3
21	*D630010B17Rik*	−2.4	21	*Ndp*	−2.3
22	*Cldn2*	−2.3	22	*Sh3rf2*	−2.2
23	*Ptger3*	−2.3	23	*Gkn3*	−2.2
24	*Batf3*	−2.3	24	*Itga10*	−2.2
25	*Trpc3*	−2.3	25	*5830408B19Rik*	−2.1
26	*BC057651*	−2.2	26	*Samd5*	−2.0
27	*Calml4*	−2.2	27	*Cox8b*	−1.9
28	*Cck*	−2.2	28	*Adamts19*	−1.9
29	*Tnnt1*	−2.2	29	*Pou3f1*	−1.9
30	*Ttr*	−2.2	30	*Ppm1e*	−1.9
43	*Foxf1*	−2.0	34	*Shisa2*	−1.8
46	*Car3*	−2.0	35	*Avpr1a*	−1.8
51	*Avpr1a*	−2.0	40	*Nkd1*	−1.8
72	*Snai1*	−1.8		*Aldh1a2*	−1.6
77	*Foxd1*	−1.8		*Thy1*	−1.4
84	*Thy1*	−1.7		*Ahr*	−1.7
85	*Bmp4*	−1.7			
115	*Aldh1a2*	−1.6			
	*Itga4*	−1.9			

AvgFC, average fold change

Using the same filtering conditions, 65 genes with reduced expression were found in IWP-2-treated ureters (Fig. 7B; Table S23; GSE282232). DAVID revealed enrichment of terms related to WNT signaling, reflecting reduced expression of components and/or targets of WNT signaling including *Axin2* (Tables 1B,2B; Table S24).

Overlapping both lists identified ten common genes (Fig. 7C; Table 3A). Four additional common genes were found with less stringent conditions of filtering (Table 3B). This suggests that SHH and WNT signaling largely activate unique sets of genes in the LP but also share a (small) set of common targets.

**Table 3.** Genes with reduced expression in both cyclopamine- and IWP-2-treated ureters.

Gene	AvgFC cyclopamine	AvgFC IWP-2
**A Top ten genes**		
*Ibsp*	−28.1	−13.8
*Notumos*	−6.1	−12.7
*Mc5r*	−4.7	−7.9
*Ndp*	−3.1	−2.3
*Sp5*	−2.6	−4.5
*Dio3*	−2.5	−1.7
*D630010B17Rik*	−2.4	−2.5
*Ptger3*	−2.3	−2.4
*Avpr1a*	−2.0	−1.8
Cox8b	−1.8	−1.9
**B Additional candidate genes**		
*Aldh1a2*	−1.6	−1.6
*Itga4*	−1.9	−2.4
*Thy1*	−1.7	−1.4
*Car3*	−2.0	−1.6
*Ahr*	–	−1.7
*Bmp4*	−1.7	–
*Foxf1*	−2.0	1.3

AvgFC, average fold change

To validate these findings and determine tissue specificity of expression, we performed RNA *in situ* hybridization analysis for individual candidates on ureter sections. We found a set of genes (*Ahr*, *Aldh1a2*, *Avpr1a*, *Axin2*, *Bmp4*, *Car3*, *Foxf1*, *Itga4*, *Ptch1*, *Thy1*) that showed specific expression in the LP at P0. Detection of expression was more difficult in cultured ureters but, in agreement with the microarray data, expression of *Aldh1a2*, *Avpr1a*, *Bmp4*, *Foxf1*, *Itga4*, *Ptch1* and *Thy1* was clearly reduced in cyclopamine-treated ureters, whereas in IWP-2-treated ureters expression of *Ahr*, *Aldh1a2* (weakly), *Avpr1a*, *Axin2*, *Itga4* and *Thy1* was reduced. Expression of *Ptch1* appeared to be slightly increased in IWP-2-treated ureters, as was *Axin2* in cyclopamine-treated ureters (Fig. 7D). We conclude that SHH and WNT signaling have distinct and common targets in the LP at perinatal stages, and may partially attenuate each other's activity.

## DISCUSSION

### LP fibrocytes are marked by expression of ALDH1A2

The lack of specific and robust cytodifferentiation markers has made the characterization of LP fibrocytes difficult (Andersson and McCloskey, 2014; Roulis and Flavell, 2016). Both in the adult bladder and intestine, LP fibrocytes were distinguished from adjacent SMCs by their localization, the lack of specific SMC markers (such as SMTN and DES) and the presence of COL1A2, VIM, CDH2 or in some cases THY1 (Kuijpers et al., 2014; Poletajew et al., 2015; Powell et al., 2011; Roulis and Flavell, 2016). We tested all of these markers in P7 ureters and found that they detected no antigen (COL1A2, THY1), stained most likely nerves (CDH2) and SMCs (DES), and marked the LP and SMCs in a patchy fashion, proving them unsuitable for LP detection in this tissue context.

It has been described that *Aldh1a2* mRNA is expressed in the LP of the E18.5 ureter (Trowe et al., 2012). However, this expression is relatively weak, it does not allow the visualization of individual cells and works poorly on adult tissue. We tested the suitability of anti-ALDH1A2 immunofluorescence for LP fibrocyte detection. In fact, ALDH1A2 recapitulated the expression of *Aldh1a2* mRNA at E18.5 and P7 in sub-urothelial mesenchymal cells, and allowed sensitive detection of individual cells by their cytoplasmic staining from E18.5 to adult stages. Importantly, ALDH1A2 expression comprised all ACTA2^−^CDH1^−^ suburothelial mesenchymal cells, indicating that LP fibrocytes are a homogenous cell population throughout development and homeostasis, and are clearly demarcated from the adjacent SMCs.

Expression of *Aldh1a2* was also reported in the sub-urothelial stroma in the developing and adult bladder (Gandhi et al., 2013), in the stroma of proximal Müllerian ducts (Nakajima et al., 2016), and in the uterine stroma (Ma et al., 2012), while expression of the related *Aldh1a3* gene was found in the developing LP of the small intestine (Niederreither et al., 2002). Therefore, expression of RA synthesizing enzymes may more generally mark the LP in different developing and adult organ contexts.

### Development of LP fibrocytes is delayed compared to that of SMCs

SMC differentiation occurs in the mesenchymal wall of the murine ureter between E15.5 to E18.5, leading to a peristaltically active tube that is able to cope with the urine that is produced in the fetal kidney from E16.5 onwards (Bohnenpoll et al., 2017a; Kurz et al., 2022b). Our histological and immunofluorescent analyses confirmed the presence of a contiguous layer of ACTA2^+^ cells in the mesenchymal wall of the ureter at E16.5 and E18.5, which only marginally gained width at postnatal stages. In contrast, only few LP fibrocytes were detected in late fetal ureters, while a contiguous LP with numerous fibrocytes embedded within a collagen-rich extracellular matrix was found at P7 and gained further width until P40. Hence, LP development is delayed compared to that of TM.

In explant cultures of P0 ureters, LP cells exhibited a proliferation rate more than double that of the adjacent muscle layer. A similar difference in proliferation rates was reported in E18.5 ureters *in vivo* (Bohnenpoll et al., 2017a). Proliferation rates of LP fibrocytes at P0 resembled those of the undifferentiated UM at E12.5, indicating that they maintain a precursor character to allow proliferative expansion of the tissue at late fetal and early postnatal stages.

*Aldh1a2* expression is found in the entire UM at E11.5 and is subsequently downregulated in the inner domain of the UM until E14.5 (Bohnenpoll et al., 2017b). Although this may support the idea that *Aldh1a2*/ALDH1A2 expression marks the aforementioned precursor state of LP fibrocytes, we deem this is unlikely given that ALDH1A2 expression in the LP is maintained at P40 when all mesenchymal cells in the ureteric wall have become quiescent (Bohnenpoll et al., 2017a).

### Differentiation and proliferation of the committed SMC/LP progenitor requires SHH and WNT signaling, whereas diversification of LP fibrocytes from SMCs is due to differential BMP4 signaling

SMCs and LP fibrocytes develop from a common *Axin2^+^Myocd^+^* precursor that resides in the inner layer of the UM at E14.5. While all of these progenitors activate the SMC differentiation program until E15.5, some cells underneath the urothelium switch off the myogenic program, express ALDH1A2 and differentiate into LP fibrocytes from E16.5 onwards (Bohnenpoll et al., 2017a). Our expression analysis of E14.5 ureters revealed that SHH, WNT, BMP4 and RA signaling are all active in the committed SMC/LP progenitor in the inner ring of the UM. We addressed which signaling activity impinges on the differentiation and lineage diversification of these committed progenitors by pharmacological pathway manipulations in explant cultures of E12.5+2 day ureters, which resemble E14.0 to E14.5 ureters *in vivo*. Inhibition of SHH or WNT signaling compromised mesenchymal expansion and abolished both SMC and LP fibrocyte differentiation, clearly showing that these two epithelial signals are required for the expansion and differentiation of the committed progenitor into both cell types but not for lineage diversification. Moreover, broad and strong activation of SHH signaling or of WNT signaling in the UM did not lead to a switch of SMCs to LP cells but to an expansion of both cell types. These findings contradict the suggestion that segregation of LP fibrocytes and SMCs from the common mesenchymal progenitor is due to differential SHH signaling, in which high doses (in the periureteral region) induce LP development, possibly by repressing the SMC fate, while low doses favor SMC differentiation (Cao et al., 2010; Yu et al., 2002).

In contrast, inhibition of BMP4 signaling compromised SMC differentiation, while differentiation of suburothelial mesenchymal cells into LP fibrocytes was not affected. This shows that positive signals for LP differentiation (SHH and WNTs) are independent of BMP4 signaling, and act onto the adjacent mesenchymal cells only. Moreover, it suggests that lineage diversification is due to a selective requirement for BMP4 signaling in the early SMC program and absence of BMP4 signaling in the LP lineage, respectively. This notion is further supported by expression analysis of the BMP4 signaling target *Id2* in ureter development. *Id2* expression was found in the inner ring of the UM at E14.5, i.e. in the committed progenitor, but was subsequently lost in the developing LP.

In E12.5 ureters co-treated with purmorphamine and NOG, the largely expanded mesenchymal cell population lost ACTA2 and gained ALDH1A2 expression. This shows that in the undifferentiated UM, i.e. in uncommitted progenitors, SHH is the activator of ALDH1A2 expression, whereas BMP4 signaling represses it.

In the undifferentiated UM, SHH signaling induces expression of the transcription factor FOXF1. FOXF1 is required but not sufficient for *Myocd* activation and SMC differentiation (Bohnenpoll et al., 2017c). In fact, FOXF1 synergizes with BMP4-induced GATA6 in this program (Kurz et al., 2022a). Absence of BMP4 signaling and GATA6 expression in the LP at perinatal and postnatal stages (this study and Kurz et al., 2022a), provides an explanation that *Foxf1* expression in this domain (this study and Fink et al., 2022), does not confer the SMC fate.

We have recently shown that loss of *Fgfr2* in the undifferentiated UM results in increased SHH and decreased BMP4 signaling, and premature activation of the LP at the expense of the SMC program (Deuper et al., 2022). These findings are entirely consistent with the conclusion of this study that LP development requires SHH (and WNT) signaling input but absence of BMP4 signaling. They suggest that lack of FGFR2-signaling-induced BMP receptor expression accounts for the loss of BMP4 signaling in LP cells.

High doses of the pan-RA receptor antagonist BMS493 in fetal ureter cultures led to a strong decrease of both SMCs and LP cells, and an increase of undifferentiated cells. This is compatible with the previously described role of RA in expanding the mesenchymal progenitors and/or inhibiting their differentiation (Bohnenpoll et al., 2017b).

### SHH and WNT signaling cooperate in postnatal LP (and SMC) development

Our histological and marker analyses showed that the LP of the murine ureter arises as clutches of ACTA2^−^ALDH1A2^+^ suburothelial mesenchymal cells at late fetal stages and develops at early postnatal stages (from P0 to P7) into a contiguous cell layer with numerous fibrocytes embedded within a collagen-rich extracellular matrix. In the first postnatal week, the LP is a target of SHH and WNT signals, and a source of BMP4 and RA. While BMP4 and RA signaling appear to be dispensable for proliferation and differentiation of LP fibrocytes and SMCs in this phase, WNT and SHH signaling are again highly relevant for these cellular processes. SHH inhibition abolished ALDH1A2 expression and affected the number of LP cells, and had a weak effect on the number of SMCs. Activation of SHH signaling slightly increased the number of LP fibrocytes but strongly expanded the number of SMCs, suggesting that SHH signals induce differentiation and proliferative expansion of LP cells, and are a limiting factor for SMC expansion. WNT signaling inhibition also reduced LP and SMC expansion but did not affect the differentiation of either cell type. Overactivation of WNT signaling did not affect the number of SMCs or LP cells, suggesting that WNTs are required for expansion of LP cells and SMCs but do not represent a limiting factor.

Together with the finding that co-inhibition of both SHH and WNT signaling reduced the number of LP cells and SMCs more strongly than individual inhibition, and that activation of one pathway can rescue the reduction of LP cells by the other pathway, we conclude that both pathways cooperate in the proliferative expansion of both LP cells and SMCs. Interestingly, inhibition of SHH signaling lowered LP proliferation, whereas WNT signaling inhibition reduced LP cell proliferation but also affected more weakly SMC proliferation We conclude that SHH and WNT pathways act in a concentration-dependent manner, with WNT signaling acting in a wider spatial range than SHH signaling.

Our transcriptional profiling uncovered forkhead transcription factor genes including *Foxf1/Foxf2*, but also *Bmp4* and *Aldh1a2* as SHH-dependent genes in the early postnatal LP. As the *Foxf1/Foxf2*-*Bmp4* module mediates SHH signaling in the undifferentiated UM at E12.5 (Bohnenpoll et al., 2017c), it is likely that SHH signaling effectors are conserved at both developmental stages. WNT signaling was required for expression of many genes, including a small set of SHH-dependent genes in the LP, showing that the two pathways have common but mostly distinct targets in this tissue. Intriguingly, expression of *Ptch1* and *Axin2* appeared to be slightly upregulated in postnatal ureters exposed to IWP-2 and cyclopamine, respectively, which may indicate a mutual dampening effect of the two pathways.

**Fig. 8.**
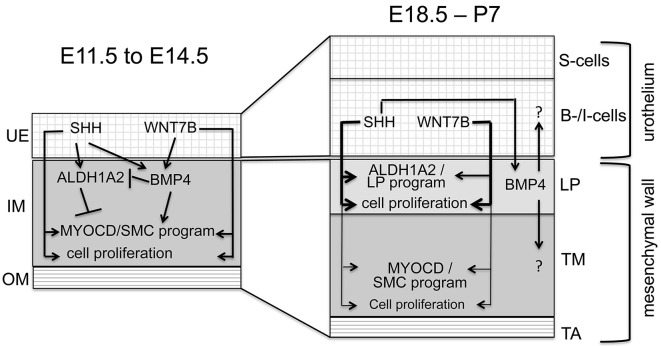
**Lineage diversification in the ureteric mesenchyme.** Scheme summarizing the signals and connected cellular processes during smooth muscle cell (SMC) and lamina propria (LP) diversification in ureter development. From E11.5 to E14.5, SHH and WNT7B signal from the ureteric epithelium (UE) to the adjacent inner mesenchyme (IM) to induce expression of ALDH1A2 and BMP4. BMP4 represses ALDH1A2 in the inner region and thus assures activation of MYOCD and of the SMC program at ∼E14.5. From ∼E18.5 to P7, epithelial SHH and WNT7B signal from B-/I-cells to the adjacent mesenchymal cells to induce proliferation and differentiation of the LP (including expression of ALDH1A2). Both signaling pathways are also combinatorially required at lower concentrations to maintain differentiation and low proliferation of SMCs (thin lines). BMP4 signals from the LP to the adjacent tunica muscularis (TM) and urothelium. The function of BMP4 signaling in these tissues is unknown. OM, outer region of the ureteric mesenchyme; TA, tunica adventitia.

Together, our study provides a molecular explanation for the development of the LP in the murine ureter (Fig. 8). SHH and WNT signals from the UE are essential for formation of the common SMC/LP progenitor in the UM, and its subsequent proliferation and differentiation into SMCs and LP fibrocytes. RA signaling expands the progenitors and inhibits their differentiation. BMP4 signals support SMC differentiation of the progenitor. It is the absence of BMP4 signaling which segregates the LP from the SMC fate. In the postnatal phase, SHH signaling maintains the differentiation and proliferation of LP cells, the latter of which is supported by WNT signals.

## MATERIALS AND METHODS

### Animals

NMRI mice were used for all experiments. For timed pregnancies, vaginal plugs detected at noon after mating were designated as E0.5. P0 was designated the day of birth. Embryos and urogenital systems were dissected in PBS, ureters for explant cultured in L-15 Leibovitz medium (BS.F1315, BIO&SELL)*.* Specimens were fixed in 4% paraformaldehyde (PFA)/PBS, transferred to methanol and stored at −20°C before immunofluorescence or *in situ* hybridization analysis.

Mice were housed in rooms with controlled light and temperature. The experiments were performed in accordance with the German Animal Welfare Legislation (TierSchG). They were approved by the local Institutional Animal Care and Research Advisory Committee and authorized by the Lower Saxony State Office for Consumer Protection and Food Safety (reference number 42500/1H).

### Organ cultures

Ureters were explanted onto a 0.4 µm pore size Transwell polyester membrane (#3450, Corning) and cultured at the air-liquid interface in DMEM/F12 (#21331020, Gibco) supplemented with 10% fetal calf serum (S0615, Merck) and 1% of concentrated stocks of penicillin/streptomycin (#15140122, Gibco), pyruvate (#11360070, Gibco), Glutamax (#35050038, Gibco), NEAA (100×, #11140035, Gibco) with 5% CO_2_ at 37°C. For pharmacological manipulation of SHH signaling, we used cyclopamine [#S1146, Selleck Chemicals (SelleckChem)] dissolved in ethanol, and purmorphamine (#S3042, SelleckChem) dissolved in DMSO. For RA signaling manipulation, we used BMS493 (#3509, Tocris; dissolved in DMSO), and RA (#0695, Tocris; dissolved in DMSO). For WNT signaling manipulation, we used IWP-2 (#S7085, SelleckChem; dissolved in DMSO) and BIO (#S7198, SelleckChem; dissolved in DMSO). For BMP4 signaling manipulation, we used NOG (#Z03205, GenScript; dissolved in MilliQ-H_2_O) and BMP4 (#5020-BP, R&D Systems; dissolved in 4 mM HCl). Medium containing the respective solvents (control) or compounds was refreshed every second day.

### Histological analysis

Embryos, urogenital systems and ureters were paraffin wax-embedded and sectioned to 5 µm (immunofluorescence, histological staining) or 10 µm (RNA *in situ* hybridization) using a Leica RM2155 or a Leica Jung RM2035 manual rotary microtome. Histological staining with Hematoxylin and Eosin (GHS332-1L, Merck/Sigma-Aldrich) and Picrosirius Red (#365548, Sigma-Aldrich) followed standard procedures (Fischer et al., 2008; Junqueira et al., 1979).

### RNA *in situ* hybridization analysis

Non-radioactive RNA *in situ* hybridization analysis was performed on whole cultured ureters and on 10 µm transversal paraffin wax sections of the proximal ureter using digoxigenin-labeled antisense riboprobes as previously described (Moorman et al., 2001; Wilkinson and Nieto, 1993).

### Immunofluorescent detection of antigens

Immunofluorescence analysis of marker proteins was performed on 5 µm paraffin wax sections with primary and secondary antibodies as listed in Table S25. The signals of ALDH1A2 and BrdU primary antibodies were amplified using the Tyramide Signal Amplification (TSA) system (NEL702001KT, PerkinElmer). Before staining, paraffin sections were deparaffinized and cooked for 15 min in antigen unmasking solution (H-3300, Vector Laboratories). Nuclei were stained with 4′,6-diamidino-2-phenylindole (DAPI, #6335.1, Carl Roth).

### Transcriptional profiling

Two independent pools each of five or six male or female P0 ureters cultured for 18 h with solvent (control) or with compounds (10 µM cyclopamine or 5 µM IWP-2) were used for microarray analysis. Total RNA from each pool was extracted using the RNeasy Micro Kit (#74004, Qiagen) and subsequently processed by the Research Core Unit Transcriptomics of Hannover Medical School for hybridization on whole Mouse Genome Oligo v2 (4×44K) microarrays (#G4846A; Agilent Technologies). Normalized expression data were filtered using Microsoft Excel. Functional enrichment analysis was performed using DAVID 6.8 web software (david.ncifcrf.gov); terms were selected based on *P*-values. Microarray data were submitted to Gene Expression Omnibus (GEO) under accession numbers GSE282232 and GSE282233.

### Cellular assays

Apoptosis was assessed by the TUNEL assay using the ApopTag Plus Fluorescein Detection Kit (S7111, Merck) on 5 μm paraffin wax sections of the proximal region of P0 ureters cultured for 18 h in the presence of inhibitors of signaling activities. Proliferation rates were assayed by detection of incorporated BrdU on 5 µm paraffin wax sections of the proximal region of P0 ureters cultured for 18 h in the presence of inhibitors of signaling activities. BrdU was added for 3 h at the end of the culture period at a concentration of 3.3 µg/ml (Bohnenpoll et al., 2017b). At least four sections of each specimen (*n*>4) were analyzed. The TM was defined by ACTA2-staining and the urothelium was marked by CDH1 expression. LP fibrocytes in between both compartments were considered negative for both markers. The BrdU-labeling index was defined as the number of BrdU-positive nuclei relative to the total number of nuclei as detected by (DAPI) counterstaining.

### Quantification and statistical analyses

For quantification of cell types, 5 µm ureter sections were co-stained for expression of ACTA2 and ALDH1A2 or for ACTA2 and CDH1. Nuclei were counterstained with DAPI. In the case of the ACTA2/ALDH1A2 co-staining, nuclei with surrounding cytoplasmic ACTA2 staining were considered as SMCs and nuclei with surrounding cytoplasmic ALDH1A2 expression were considered as LP cells. In the case of the ACTA2/CDH1 co-staining, LP cells were recognized as nuclei lying between the ACTA2^+^ SMCs and CDH1^+^ urothelium. Cells were manually counted using the counting tool in Adobe Photoshop CS4 extended.

For the measurement of the LP thickness, sections were co-stained with antibodies against ACTA2 and CDH1. LP cells were recognized as nuclei lying between the ACTA2^+^ SMCs and CDH1^+^ urothelium. Using the measuring tool of the Fiji/ImageJ software, the distance between the ACTA2 and CDH1 staining was measured at five distinct positions per section and averaged. The measurements were converted to micrometers for statistical analysis.

Statistical analysis was performed using GraphPad Prism Version 7a. The Shapiro-Wilk normality test was performed to analyze the distribution within the different data groups [normal distribution (parametric), *P*-values≥0.05]. The *F*-test of equality of variances was used to analyze the variances of two data groups (equal variances, *P*-values≥0.05). Depending on the results of the Shapiro-Wilk and the *F*-test, different tests were used to compare two different groups. To compare two groups with normal distribution and equal variances the unpaired two-tailed *t*-test was applied. The two-tailed Welch's *t*-test was used if two groups with normal distribution had unequal variances. The two-tailed Mann–Whitney *U*-test was used to compare two groups with abnormal distribution (non parametric). Results were shown as means with standard deviation. A *P*-value of <0.05 was considered as statistically significant (*), a *P*-value of *P*<0.01 as highly significant (**) and *P*-values of *P*<0.001 (***) as extremely significant.

### Image documentation

Sections were photographed using a DM5000 microscope (Leica Camera) with a Leica DFC300FX digital camera, or a Leica DMI6000B microscope with a Leica DFC350FX digital camera. All images were assembled into figures in Adobe Photoshop 2020, CS3 or CS4.

## Supplementary Material



10.1242/develop.204214_sup1 Supplementary information

Table S1. The number of LPs cells and the thickness of the LP cell layer increase at postnatal stages (relates to Fig. 1E,F). P0, P7 and P40 *in vivo* ureters were sectionned and analysed for expressino of the smooth muscle cell marker ACTA2 and the urothelial marker CDH1. (**A**) The total number of ACTA2+ smooth muscle cells and ACTA2- CDH1- suburothelial mesenchymal (LP) cells was determined as was the ratio of the two cell types. (**B**) To determine the thickness of the ACTA2- CDH1- cell layer, the software ImageJ/Fiji was used. Pixels were converted in μm by measuring the scale bar of five pictures and the means were used to determine the pixel-μm factor. With this factor each measurement was converted into μm.

Table S2. Effect of increasing concentrations of cyclopamine on lamina propria cells in cultures of E12.5 ureters (A relates to Fig. 3A and B to Fig. S6A). Ureters were explanted at E12.5 and cultured for 2 days without cyclopamine and for a further 6 days with increasing concentrations of cyclopamine. (**A**) Cultures were then processed for expression of the smooth muscle cell (SMC) marker ACTA2 and the lamina propria (LP) marker ALDH1A2, and the total number of cells and the ratio of ACTA2+ALDH1A2- SMCs, ACTA2- ALDH1A2+ LP fibrocytes, and ACTA2-ALDH1A2- “undifferentiated” LP cells were determined. (**B**) Cultures were additionally stained for the SMC marker ACTA2 and the urothelial marker CDH1 and the total number of cells and the ratio of ACTA2+CDH1- SMCs and ACTA2-CDH1- LP cells were determined.

Table S3. Effect of increasing concentrations of purmorphamine on lamina propria cells in cultures of E12.5 ureters (A relates to Fig. 3A and B to Fig. S6A). Ureters were explanted at E12.5 and cultured for 2 days without purmorphamine and for a further 6 days with increasing concentrations of purmorphamine. (**A**) Cultures were then processed for expression of the smooth muscle cell (SMC) marker ACTA2 and the lamina propria (LP) marker ALDH1A2, and the total number of cells and the ratio of ACTA2+ALDH1A2- SMCs, ACTA2-ALDH1A2+ LP fibrocytes, and ACTA2-ALDH1A2- “undifferentiated” LP cells were determined. (**B**) Cultures were additionally stained for the SMC marker ACTA2 and the urothelial marker CDH1 and the total number of cells and the ratio of ACTA2+CDH1- SMCs and ACTA2-CDH1- LP cells were determined.

Table S4. Effect of increasing concentrations of IWP-2 on lamina propria cells in cultures of E12.5 ureters (A relates to Fig. 3B and B to Figure S6B). Ureters were explanted at E12.5 and cultured for 2 days without IWP-2 and for a further 6 days with increasing concentrations of IWP-2. (**A**) Cultures were then processed for expression of the smooth muscle cell (SMC) marker ACTA2 and the lamina propria (LP) marker ALDH1A2, and the total number of cells and the ratio of ACTA2+ALDH1A2- SMCs, ACTA2-ALDH1A2+ LP fibrocytes, and ACTA2-ALDH1A2- “undifferentiated” LP cells were determined. (**B**) Cultures were additionally stained for the SMC marker ACTA2 and the urothelial marker CDH1 and the total number of cells and the ratio of ACTA2+CDH1- SMCs and ACTA2-CDH1- LP cells were determined.

Table S5. Effect of increasing concentrations of BIO on lamina propria cells in cultures of E12.5 ureters (A relates to Fig. 3B and B to Fig. S6B). Ureters were explanted at E12.5 and cultured for 2 days without BIO and for a further 6 days with increasing concentrations of BIO. (**A**) Cultures were then processed for expression of the smooth muscle cell (SMC) marker ACTA2 and the lamina propria (LP) marker ALDH1A2, and the total number of cells and the ratio of ACTA2+ALDH1A2- SMCs, ACTA2-ALDH1A2+ LP fibrocytes, and ACTA2-ALDH1A2- “undifferentiated” LP cells were determined. (**B**) Cultures were additionally stained for the SMC marker ACTA2 and the urothelial marker CDH1 and the total number of cells and the ratio of ACTA2+CDH1- SMCs and ACTA2- CDH1- LP cells were determined.

Table S6. Effect of increasing concentrations of NOG on lamina propria cells in cultures of E12.5 ureters (A relates to Fig. 3C and B to Fig. S6C). Ureters were explanted at E12.5 and cultured for 2 days without NOG and for a further 6 days with increasing concentrations of NOG. (**A**) Cultures were then processed for expression of the smooth muscle cell (SMC) marker ACTA2 and the lamina propria (LP) marker ALDH1A2, and the total number of cells and the ratio of ACTA2+ALDH1A2- SMCs, ACTA2-ALDH1A2+ LP fibrocytes, and ACTA2-ALDH1A2- “undifferentiated” LP cells were determined. (**B**) Cultures were additionally stained for the SMC marker ACTA2 and the urothelial marker CDH1 and the total number of cells and the ratio of ACTA2+CDH1- SMCs and ACTA2- CDH1- LP cells were determined.

Table S7. Effect of increasing concentrations of BMP4 on lamina propria cells in cultures of E12.5 ureters (A relates to Fig. 3C and B to Fig. S6C). Ureters were explanted at E12.5 and cultured for 2 ys without BMP4 and for a further 6 days with increasing concentrations of BMP4. (**A**) Cultures were then processed for expression of the smooth muscle cell (SMC) marker ACTA2 and the lamina propria (LP) marker ALDH1A2, and the total number of cells and the ratio of ACTA2+ALDH1A2- SMCs, ACTA2-ALDH1A2+ LP fibrocytes, and ACTA2-ALDH1A2- “undifferentiated” LP cells were determined. (**B**) Cultures were additionally stained for the SMC marker ACTA2 and the urothelial marker CDH1 and the total number of cells and the ratio of ACTA2+CDH1- SMCs and ACTA2-CDH1- LP cells were determined.

Table S8. Effect of increasing concentrations of BMS493 on lamina propria cells in cultures of E12.5 ureters (A relates to Fig. 3D and B to Fig. S6D). Ureters were explanted at E12.5 and cultured for 2 days without BMS493 and for a further 6 days with increasing concentrations of BMS493. (**A**) Cultures were then processed for expression of the smooth muscle cell (SMC) marker ACTA2 and the lamina propria (LP) marker ALDH1A2, and the total number of cells and the ratio of ACTA2+ALDH1A2- SMCs, ACTA2-ALDH1A2+ LP fibrocytes, and ACTA2-ALDH1A2- “undifferentiated” LP cells were determined. (**B**) Cultures were additionally stained for the SMC marker ACTA2 and the urothelial marker CDH1 and the total number of cells and the ratio of ACTA2+CDH1- SMCs and ACTA2-CDH1- LP cells were determined.

Table S9. Effect of increasing concentrations of retinoic acid (RA) on lamina propria cells in cultures of E12.5 ureters (A relates to Fig. 3D and B to Fig. S6D). Ureters were explanted at E12.5 and cultured for 2 days without retinoic acid (RA) and for a furth 6 days with increasing concentrations of RA. (**A**) Cultures were then processed for expression of the smooth muscle cell (SMC) marker ACTA2 and the lamina propria (LP) marker ALDH1A2, and the total number of cells and the ratio of ACTA2+ALDH1A2- SMCs, ACTA2-ALDH1A2+ LP fibrocytes, and ACTA2-ALDH1A2- “undifferentiated” LP cells were determined. (**B**) Cultures were additionally stained for the SMC marker ACTA2 and the urothelial marker CDH1 and the total number of cells and the ratio of ACTA2+CDH1- SMCs and ACTA2- CDH1- LP cells were determined.

Table S10. Effect of increasing concentrations of cyclopamine on lamina propria cells in cultures of P0 ureters (A relates to Fig. 4A and B to Fig. S7A). Ureters were explanted at P0 and cultured for 6 days with increasing concentrations of cyclopamine. (**A**) Cultures were then processed for expression of the smooth muscle cell (SMC) marker ACTA2 and the lamina propria (LP) marker ALDH1A2, and the total number of cells and the ratio of ACTA2+ALDH1A2- SMCs, ACTA2-ALDH1A2+ LP fibrocytes, and ACTA2-ALDH1A2- “undifferentiated” LP cells were determined. (**B**) Cultures were additionally stained for the SMC marker ACTA2 and the urothelial marker CDH1 and the total number of cells and the ratio of ACTA2+CDH1- SMCs and ACTA2-CDH1- LP cells were determined.

Table S11. Effect of increasing concentrations of purmorphamine on lamina propria cells in cultures of P0 ureters (A relates to Fig. 4A and B to Fig. S7A). Ureters were explanted at P0 and cultured for 6 days with increasing concentrations of purmorphamine. (**A**) Cultures were then processed for expression of the smooth muscle cell (SMC) marker ACTA2 and the lamina propria (LP) marker ALDH1A2, and the total number of cells and the ratio of ACTA2+ALDH1A2- SMCs, ACTA2-ALDH1A2+ LP fibrocytes, and ACTA2-ALDH1A2- “undifferentiated” LP cells were determined. (**B**) Cultures were additionally stained for the SMC marker ACTA2 and the urothelial marker CDH1 and the total number of cells and the ratio of ACTA2+CDH1- SMCs and ACTA2-CDH1- LP cells were determined.

Table S12. Effect of increasing concentrations of IWP-2 on lamina propria cells in cultures of P0 ureters (A relates to Fig. 4B and B to Fig. S7B) Ureters were explanted at P0 and cultured for 6 days with increasing concentrations of IWP-2. (**A**) Cultures were then processed for expression of the smooth muscle cell (SMC) marker ACTA2 and the lamina propria (LP) marker ALDH1A2, and the total number of cell and the ratio of ACTA2 +ALDH1A2- SMCs, ACTA2-ALDH1A2+ LP fibrocytes, and ACTA2-ALDH1A2- “undifferentiated” LP cells were determined. (**B**) Cultures were additionally stained for the SMC marker ACTA2 and the urothelial marker CDH1 and the total number of cells and the ratio of ACTA2+CDH1- SMCs and ACTA2-CDH1- LP cells were determined.

Table S13. Effect of increasing concentrations of BIO on lamina propria cells in cultures of P0 ureters (A relates to Fig. 4B and B to Fig. S7B). Ureters were explanted at P0 and cultured for 6 days with increasing concentrations of BIO. (**A**) Cultures were then processed for expression of the smooth muscle cell (SMC) marker ACTA2 and the lamina propria (LP) marker ALDH1A2, and the total number of cells and the ratio of ACTA2 +ALDH1A2- SMCs, ACTA2-ALDH1A2+ LP fibrocytes, and ACTA2-ALDH1A2- “undifferentiated” LP cells were determined. (**B**) Cultures were additionally stained for the SMC marker ACTA2 and the urothelial marker CDH1 and the total number of cells and the ratio of ACTA2+CDH1- SMCs and ACTA2-CDH1- LP cells were determined.

Table S14. Effect of increasing concentrations of NOG on lamina propria cells in cultures of P0 ureters (A relates to Fig. 4C and B to Fig. S7C). Ureters were explanted at P0 and cultured for 6 days with increasing concentrations of NOG. (**A**) Cultures were then processed for expression of the smooth muscle cell (SMC) marker ACTA2 and the lamina propria (LP) marker ALDH1A2, and the total number of cells and the ratio of ACTA2 +ALDH1A2- SMCs, ACTA2-ALDH1A2+ LP fibrocytes, and ACTA2-ALDH1A2- “undifferentiated” LP cells were determined. (**B**) Cultures were additionally stained for the SMC marker ACTA2 and the urothelial marker CDH1 and the total number of cells and the ratio of ACTA2+CDH1- SMCs and ACTA2-CDH1- LP cells were determined.

Table S15. Effect of increasing concentrations of BMP4 on lamina propria cells in cultures of P0 ureters (A relates to Fig. 4C and B to Fig. S7C) Ureters were explanted at P0 and cultured for 6 days with increasing concentrations of BMP4. (**A**) Cultures were then processed for expression of the smooth muscle cell (SMC) marker ACTA2 and the lamina propria (LP) marker ALDH1A2, and the total number of cells and the ratio of ACTA2 +ALDH1A2- SMCs, ACTA2-ALDH1A2+ LP fibrocytes, and ACTA2-ALDH1A2- “undifferentiated” LP cells were determined. (**B**) Cultures were additionally stained for the SMC marker ACTA2 and the urothelial marker CDH1 and the total number of cells and the ratio of ACTA2+CDH1- SMCs and ACTA2-CDH1- LP cells were determined.

Table S16. Effect of increasing concentrations of BMS493 on lamina propria cells in cultures of P0 ureters (A relates to Figure 4D and B to Figure S7D) Ureters were explanted at P0 and cultured for 6 days with increasing concentrations of BMS493. (**A**) Cultures were then processed for expression of the smooth muscle cell (SMC) marker ACTA2 and the lamina propria (LP) marker ALDH1A2, and the total number of cells and the ratio) of ACTA2+ALDH1A2- SMCs, ACTA2-ALDH1A2+ LP fibrocytes, and ACTA2-ALDH1A2- “undifferentiated” LP cells were determined. (**B**) Cultures were additionally stained for the SMC marker ACTA2 and the urothelial marker CDH1 and the total number of cells and the ratio of ACTA2+CDH1- SMCs and ACTA2-CDH1- LP cells were determined.

Table S17. Effect of increasing concentrations of retinoic acid (RA) on on lamina propria cells in cultures of P0 ureters (A relates to Figure 4D and B to Figure S7D). Ureters were explanted at P0 and cultured for 6 days with increasing concentrations of retinoic acid (RA). (**A**) Cultures were then processed for expression of the smooth muscle cell (SMC) marker ACTA2 and the lamina propria (LP) marker ALDH1A2, and the total number of cells (nd the ratio (ACTA2+ALDH1A2- SMCs, ACTA2-ALDH1A2+ LP fibrocytes, and ACTA2-ALDH1A2- “undifferentiated” LP cells were determined. (**B**) Cultures were additionally stained for the SMC marker ACTA2 and the urothelial marker CDH1 and the total number of cells and the ratio of ACTA2+CDH1- SMCs and ACTA2-CDH1- LP cells were determined.

Table S18. Effect of combination of SHH and WNT activators and inhibitors on lamina propria cells in cultures of P0 ureters (A relates to Fig. 5B and B relates to Fig. 5C). Ureters were explanted at P0 and cultured for 6 days with different combinations of SHH and WNT activators (purmorphamine and BIO) and inhibitors (cyclopamine and IWP-2). (**A**) Cultures were then processed for expression of the smooth muscle cell (SMC) marker ACTA2 and the lamina propria (LP) marker ALDH1A2, and the total number of cells and the ratio of ACTA2+ALDH1A2- SMCs, ACTA2-ALDH1A2+ LP fibrocytes, and ACTA2-ALDH1A2- “undifferentiated” LP cells were determined. (**B**) Cultures were additionally stained for the SMC marker ACTA2 and the urothelial marker CDH1 and the total number of cells and the ratio of ACTA2+CDH1- SMCs and ACTA2-CDH1- LP cells were determined.

Table S19. Effect of combination of SHH activation and BMP4 inhibition on lamina propria cells in cultures of E12.5, E12.5+2d and P0 ureters (relates to Figure 5E). Ureters were explanted at E12.5 and P0. The E12.5 ureters were cultured for 2 days without and then cultured for 6 days with SHH activator purmorphamine and the BMP4 inhibitor NOG. P0 ureters were directly cultured with SHH activator purmorphamine and the BMP4 inhibitor NOGGIN. (**A**) Cultures were then processed for expression of the smooth muscle cell (SMC) marker ACTA2 and the lamina propria (LP) marker ALDH1A2, and the total number of cells and the ratio of ACTA2+ALDH1A2- SMCs, ACTA2-ALDH1A2+ LP fibrocytes, and ACTA2- ALDH1A2- “undifferentiated” LP cells were determined.

Table S20. Statistical analysis of mesenchymal cell proliferation in 18 h cultures of P0 ureter explants treated with individual signaling pathway inhibitors and BrdU (relates to Fig. 6C and 6D). After the 18 hours culture period ureters were fixed, embedded in paraffine- wax and sectioned to 5-μm before performing co-immunofluorescence analysis for ACTA2 and CDH1 expression and BrdU insertion. The total number of ACTA2+ smooth muscle cells (SMCs) and ACTA2- CDH1- lamina propria (LP) cells was determined, as was the ratio of BrdU+ ACTA2+ CDH- SMCs and BrdU+ ACTA2- CDH- LP fibrocytes.

Table S21. List of genes with decreased expression in micoroarrays of explants of P0 ureters treated for 18 h with 10 μM cyclopamine (relates to Fig. 7A and 7C). Shown are the gene names, the intensity of the two control and mutant ureter samples, the individual and the average (avg) fold change (FC). Genes which are also dowregulated in P0 ureters treated with 5 μM IWP-2 are marked in red (significant in all pools), and in green (significant in one of the two pools).

Table S22. Functional annotation by DAVID for genes with decreased expression in P0 ureters treated for 18 h with 10 μM of the SHH signaling inhibitor cyclopamine (relates to Fig. 7B).

Table S23. List of genes with decreased expression in micoroarrays of explants of P0 ureters treated for 18 h with 5 μM IWP-2 (relates to Fig. 7D and 7F). Shown are the gene names, the intensity of the two control and mutant ureter samples, the individual and the average (avg) fold change (FC). Genes which are also dowregulated in P0 ureters treated with 10 μM cyclopamine are marked in red (significant in all pools), and in green (significant in one of the two pools).

Table S24. Functional annotation by DAVID for genes with decreased expression in P0 ureters treated for 18 h with 5 μM of the WNT signaling inhibitor IWP-2 (relates to Fig. 7E).

Table S25. List of antibodies for immunofluoresent detection of antigens on paraffin wax sections (relates to materials and methods).
