# Supplementary material for: Interplay of SHH, WNT and BMP4 signaling regulates the development of the lamina propria in the murine ureter
Source: Development. 2025 Feb 6;152(3):DEV204214. doi: 10.1242/dev.204214 (PMC11829765; doi:10.1242/dev.204214)
Supplement: Supplementary information [file develop-152-204214-s1.pdf]

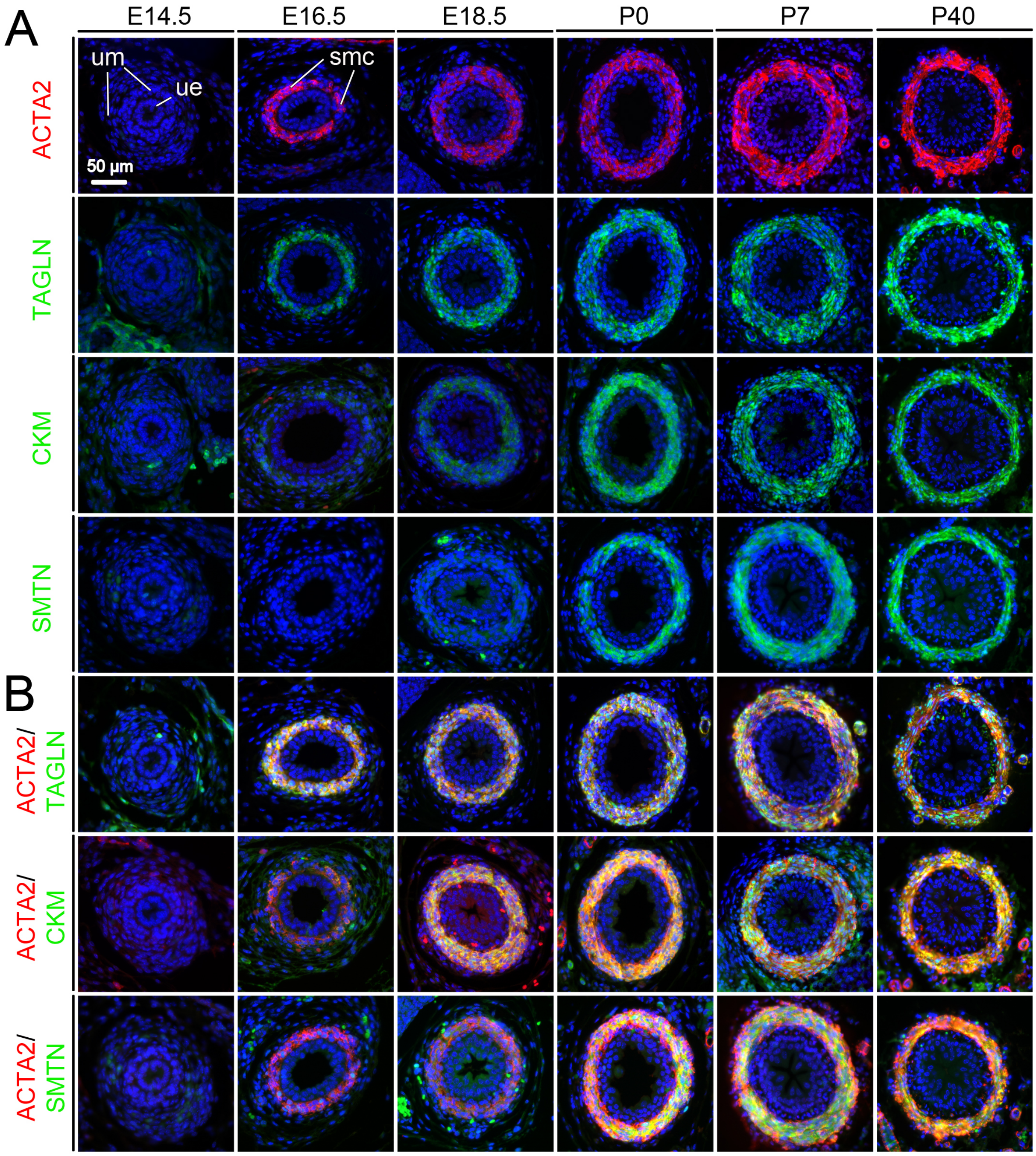

Fig. S1. Immunofluorescence analysis of expression of smooth muscle cell markers in the murine ureter from E14.5 to P40. Immunofluorescence analysis of individual expression of actin alpha 2, smooth muscle, aorta (ACTA2), transgelin (TAGLN), creatine kinase, muscle (CKM) and smoothelin (SMTN) (**A**) and of coexpression of ACTA2 with TAGLN, CKM or SMTN (**B**) on transverse sections of the proximal ureter in wildtype mice at fetal stages (E14.5, E16.5, E18.5) and at postnatal stages (P0, P7, P40). *n*=5 for each assay and each stage. smc, smooth muscle cells; ue, ureteric epithelium; um, ureteric mesenchyme.

Development • Supplementary information

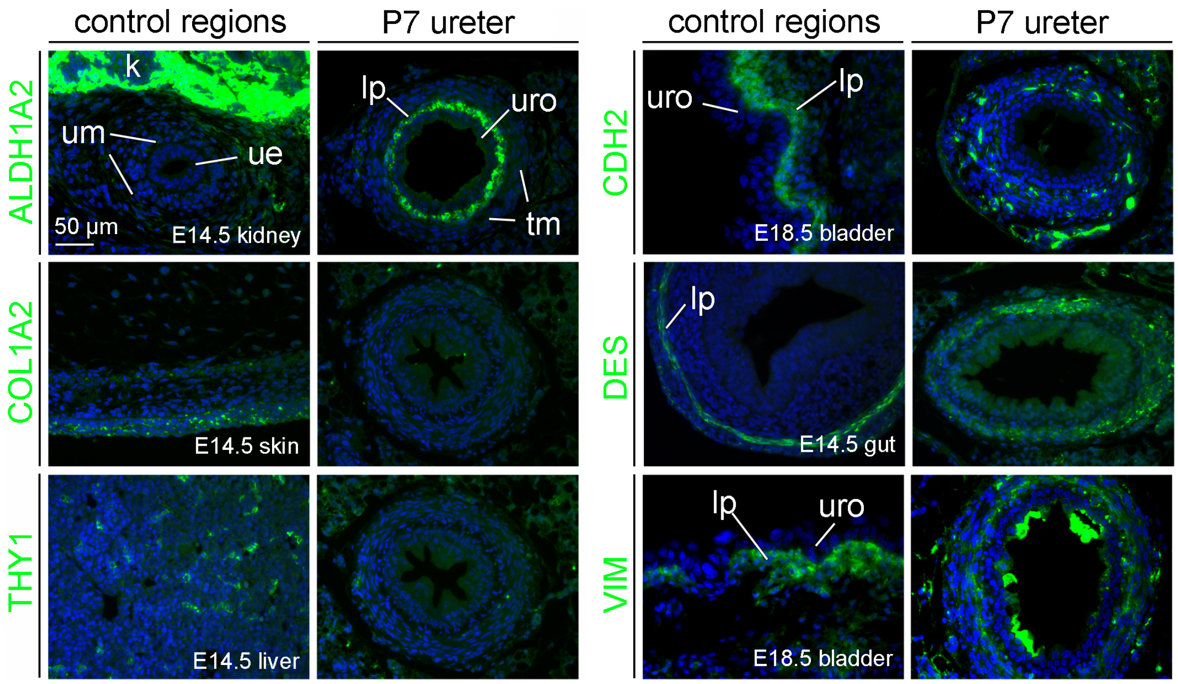

Fig. S2. Immunofluorescence analysis of markers for lamina propria fibrocytes in the murine ureter at P7. Immunofluorescence analysis of expression of aldehyde dehydrogenase family 1, subfamily A2 (ALDH1A2), cadherin 2 (CDH2, aka N-cadherin), collagen type I alpha 2 (COL1A2), desmin (DES), thymus cell antigen 1 theta (THY1) and vimentin (VIM) on sections of various control regions of embryos at E14.5 or E18.5, and on transverse sections of the proximal ureter in wildtype mice at P7. Note that ALDH1A2 reliably stains the cytoplasm of lamina propria fibrocytes in P7 ureters. COL1A2 and THY1 show no staining in the ureter, whereas CDH2 stains nerves. DES stains smooth muscle cells, whereas VIM stains superficial cells and the lamina propria albeit in a very patchy fashion. *n*=5 for each assay and each stage. k, kidney; lp, lamina propria; tm, tunica muscularis; ue, ureteric epithelium; um, ureteric mesenchyme; uro, urothelium.

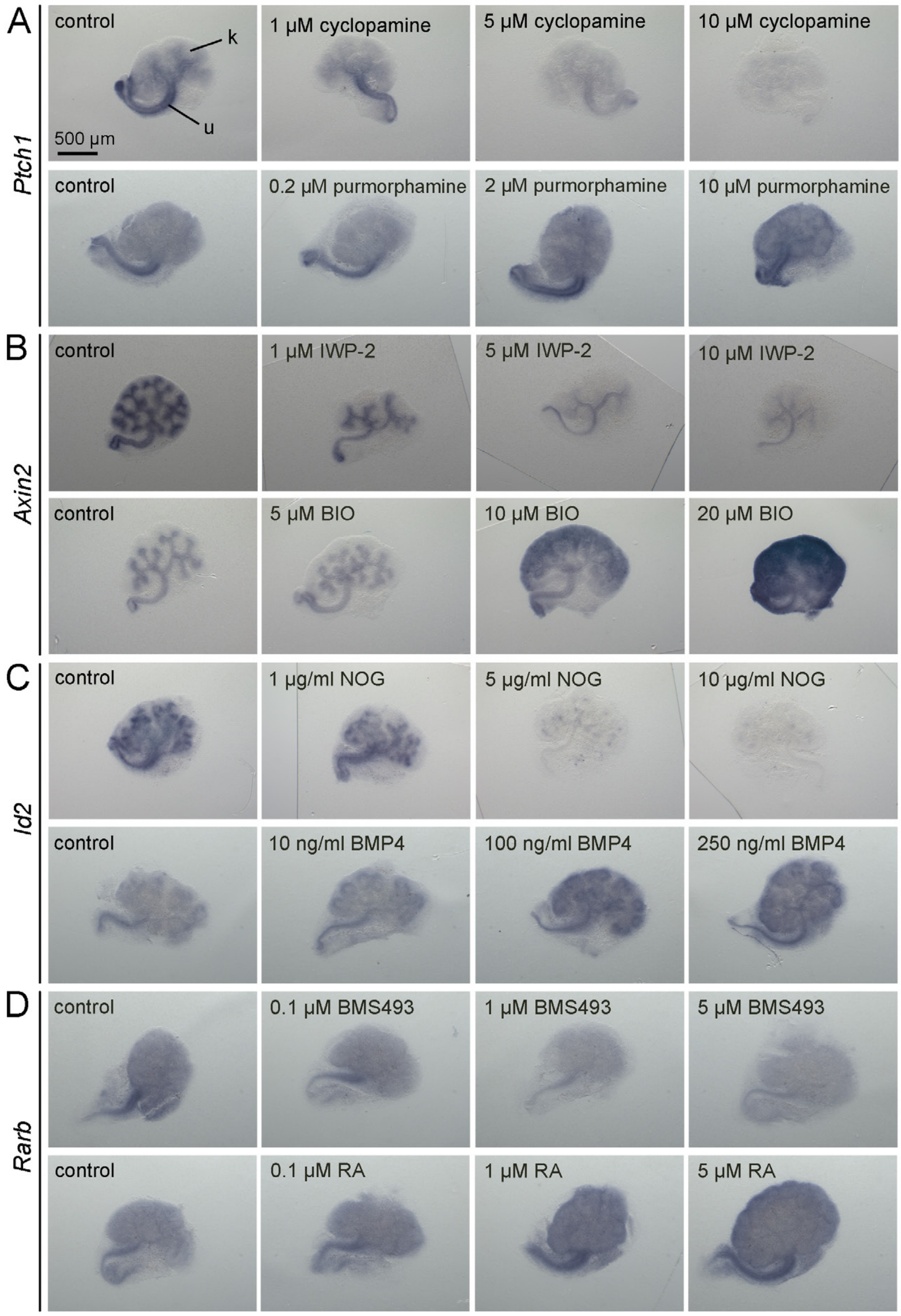

Fig. S3. Titration of pharmacological inhibitors and activators of signaling pathways in cultured explants of E12.5 ureters. (**A-D**) Embryonic ureters and kidneys were explanted at E12.5 and cultured for 18 h in the presence of inhibitors or activators of signaling activities before they were processed for RNA *in situ* hybridization analysis for expression of target genes of these signaling activities. (**A**) SHH signaling, read-out: *Ptch1*; inhibitor: cyclopamine (1-10 μ M), activator: purmorphamine (0.2-10 μ M). (**B**) WNT signaling, read-out: *Axin2*; inhibitor: IWP-2 (1-10 μM), activator: BIO (5-20 μ M). (**C**) BMP4 signaling, read-out: *Id2;* inhibitor: NOG (1-10 μ g/ml), activator: BMP4 (10-250 ng/ml). (**D**) Retinoic acid signaling, read-out: *Rarb*, inhibitor: BMS493 (0.1-5 μM), activator: retinoic acid (RA, 0.1-5 μM). *n*=5 for each assay. k, kidney; u, ureter.

Development • Supplementary information

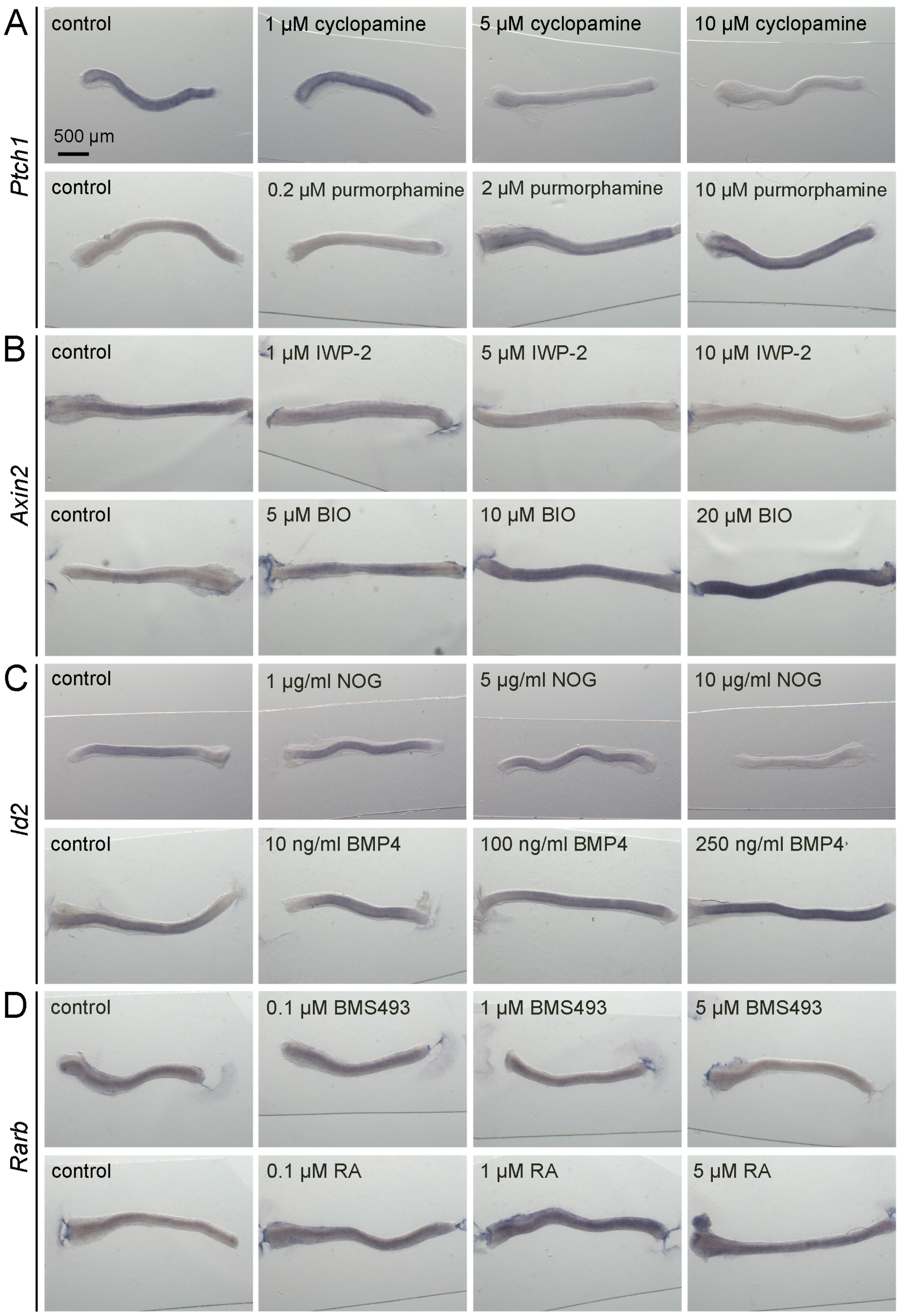

Fig. S4. Titration of pharmacological inhibitors and activators of signaling pathways in cultured explants of P0 ureters. (**A-D**) Ureters were explanted at P0 and cultured for 18 h in the presence of inhibitors or activators of signaling activities before they were processed for RNA *in situ* hybridization analysis for expression of target genes of these signaling activities. (**A**) SHH signaling, read-out: *Ptch1*; inhibitor: cyclopamine (1-10 µ M), activator: purmorphamine (0.2-10 µM). (**B**) WNT signaling, read-out: *Axin2*; inhibitor: IWP-2 (1-10 µM), activator: BIO (5-20 µ M). (**C**) BMP4 signaling, read-out: *Id2;* inhibitor: NOG (1-10 µ g/ml), activator: BMP4 (10-250 ng/ml). (**D**) Retinoic acid signaling, read-out: *Rarb*, inhibitor: BMS493 (0.1-5 µM), activator: retinoic acid (RA, 0.1-5 µM). *n*=5 for each assay.

Development • Supplementary information

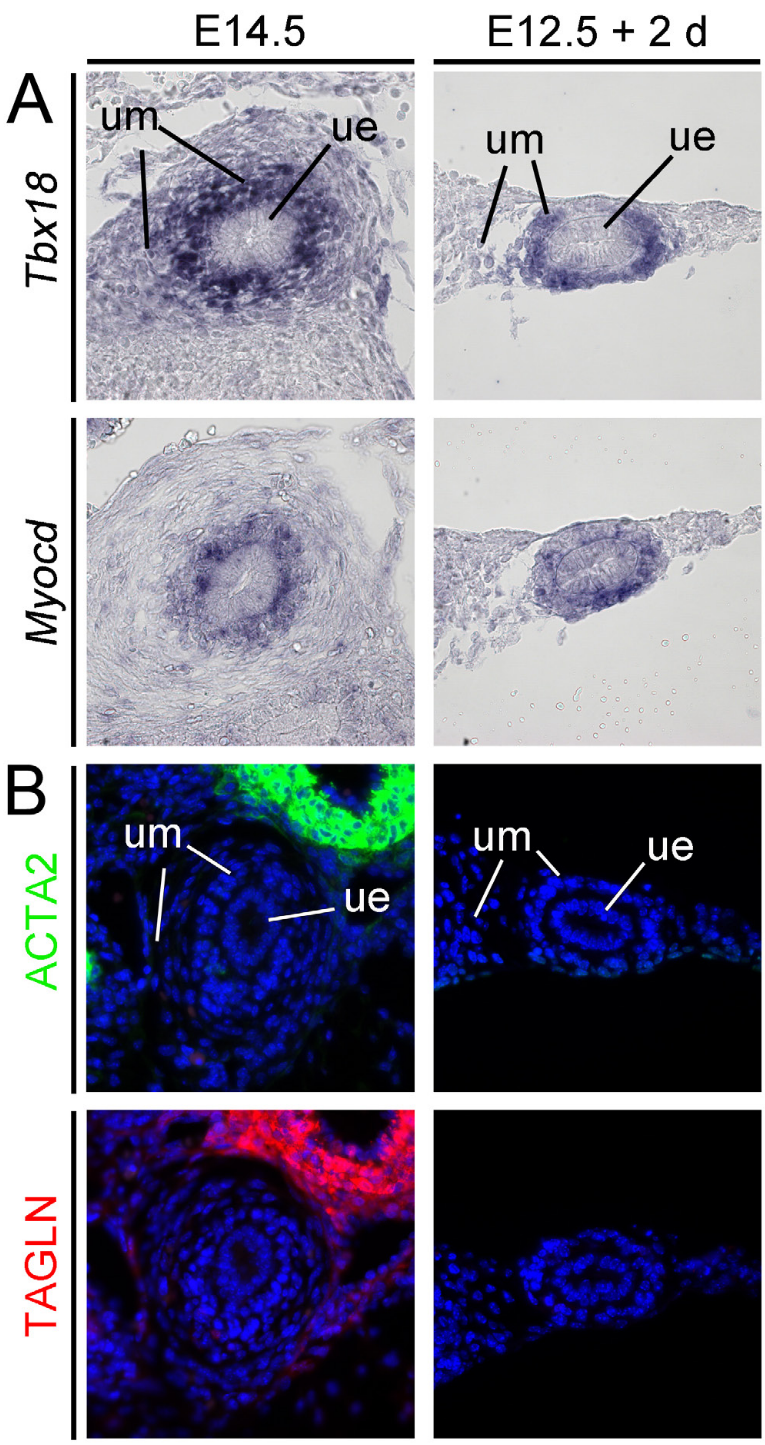

Fig. S5. Explants of E12.5 ureters cultured for 2 days reach a developmental stage similar to E14.5 ureters *in vivo*. Sections from the proximal region of E12.5 ureters cultured for 2 days and of E14.5 ureters were analyzed for expression of *Tbx18* and *Myocd* by RNA *in situ* hybridization (**A**) and for expression of ACTA2 and TAGLN by immunofluorescence (**B**). Expression of *Tbx18* and *Myocd* is found in the ureteric mesenchyme at E14.5 *in vivo*. ACTA2 and TAGLN are not yet expressed at this stage. Expression of *Tbx18* and *Myocd* and absence of expression of ACTA2 and TAGLN in E12.5+2 day ureter explants confirms that they reach a stage similar to E14.5 *in vivo*. ue, ureteric epithelium; um, ureteric mesenchyme.

Development • Supplementary information

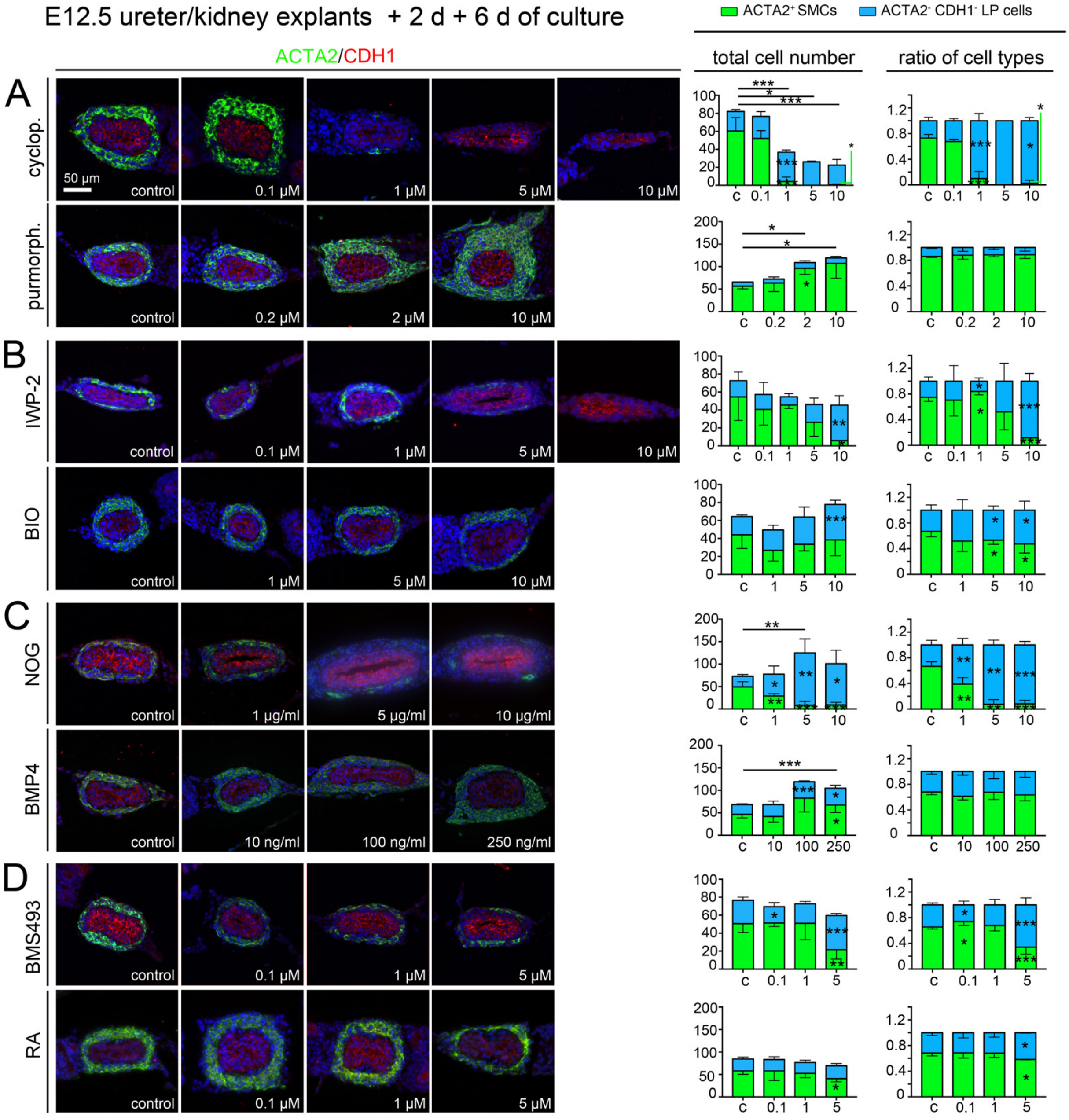

Fig. S6. Pharmacological analysis of signaling pathways in the fetal development of the lamina propria. (**A-D**) Embryonic ureters and kidneys were explanted at E12.5 and cultured for 2 days before they were incubated for another 6 days with increasing concentrations of pharmacological inhibitors or activators of SHH signaling [0.1-10 μM cyclopamine (cyclop.), 0.2-10 μM purmorphamine (purmorph.)] (**A**), WNT signaling [0.1-10 μM IWP-2, 1-10 μM BIO] (**B**), BMP4 signaling [1-10 μg/ml NOG, 10-250 ng/ml BMP4] (**C**), and retinoic acid (RA) signaling [0.1-5 μM BMS493, 0.1-5 μM retinoic acid (RA)] (**D**). Cultures were then processed for expression of the smooth muscle cell (SMC) marker ACTA2 and the urothelial marker CDH1 (left panel), and the total number of cells and the ratio of $ACTA2^{+}CDH1^{-}$ SMCs (green) and $ACTA2^{-}CDH1^{-}$ LP (mesenchymal) cells (blue) were determined. *n*=5 for each assay. For numbers and statistics (two-tailed Student's t-test, Welch's t-test or Mann-Whitney U test) see Table S2B-S9B. *P<0.05; **P<0.01; ***P<0.001. c, control.

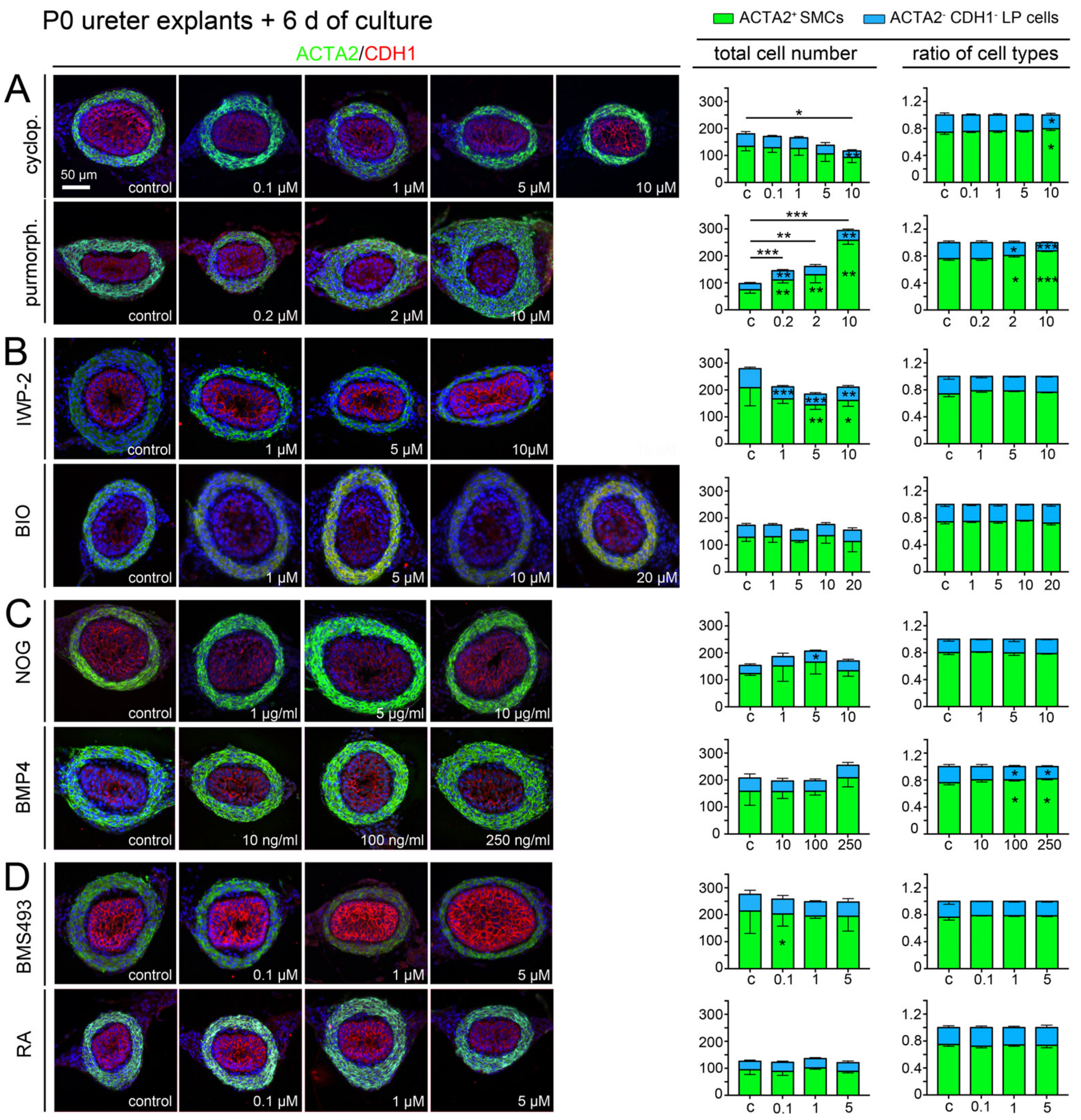

Fig. S7. Pharmacological analysis of signaling pathways in the postnatal development of the lamina propria. (**A-D**) Embryonic ureters and kidneys were explanted at P0 and cultured for 6 days in the presence of increasing concentrations of pharmacological inhibitors or activators of SHH signaling [0.1-10 µM cyclopamine (cyclop.), 0.2-10 µM purmorphamine (purmorph.)] (**A**), WNT signaling [1-10 µM IWP-2, 1-20 µM BIO] (**B**), BMP4 signaling [1-10 µg/ml NOG, 10-250 ng/ml BMP4] (**C**), and retinoic acid (RA) signaling [0.1-5 µM BMS493, 0.1-5 µM retinoic acid (RA)] (**D**). Cultures were then processed for expression of the smooth muscle cell (SMC) marker ACTA2 and the urothelial marker CDH1 (left panel), and the total number of cells and the ratio of $ACTA2^{+}CDH1^{-}$ SMCs (green) and $ACTA2^{-}CDH1^{-}$ LP (mesenchymal) cells (blue) were determined. *n*=5 for each assay. For numbers and statistics (two-tailed Student's t-test, Welch's t-test or Mann-Whitney U test) see Table S10B-S17B. *P<0.05; **P<0.01; ***P<0.001. c, control.

Development • Supplementary information

Table S1. The number of LPs cells and the thickness of the LP cell layer increase at postnatal stages (relates to Fig. 1E,F). P0, P7 and P40 *in vivo* ureters were sectionned and analysed for expressino of the smooth muscle cell marker ACTA2 and the urothelial marker CDH1. (**A**) The total number of ACTA2+ smooth muscle cells and ACTA2- CDH1- suburothelial mesenchymal (LP) cells was determined as was the ratio of the two cell types. (**B**) To determine the thickness of the ACTA2- CDH1- cell layer, the software ImageJ/Fiji was used. Pixels were converted in µm by measuring the scale bar of five pictures and the means were used to determine the pixel-µm factor. With this factor each measurement was converted into µm.

Available for download at
https://journals.biologists.com/dev/article-lookup/doi/10.1242/dev.204214#supplementary-data

Table S2. Effect of increasing concentrations of cyclopamine on lamina propria cells in cultures of E12.5 ureters (A relates to Fig. 3A and B to Fig. S6A). Ureters were explanted at E12.5 and cultured for 2 days without cyclopamine and for a further 6 days with increasing concentrations of cyclopamine. (**A**) Cultures were then processed for expression of the smooth muscle cell (SMC) marker ACTA2 and the lamina propria (LP) marker ALDH1A2, and the total number of cells and the ratio of ACTA2+ALDH1A2- SMCs, ACTA2-ALDH1A2+ LP fibrocytes, and ACTA2-ALDH1A2- “undifferentiated” LP cells were determined. (**B**) Cultures were additionally stained for the SMC marker ACTA2 and the urothelial marker CDH1 and the total number of cells and the ratio of ACTA2+CDH1- SMCs and ACTA2-CDH1- LP cells were determined.

Available for download at
https://journals.biologists.com/dev/article-lookup/doi/10.1242/dev.204214#supplementary-data

Table S3. Effect of increasing concentrations of purmorphamine on lamina propria cells in cultures of E12.5 ureters (A relates to Fig. 3A and B to Fig. S6A). Ureters were explanted at E12.5 and cultured for 2 days without purmorphamine and for a further 6 days with increasing concentrations of purmorphamine. (**A**) Cultures were then processed for expression of the smooth muscle cell (SMC) marker ACTA2 and the lamina propria (LP) marker ALDH1A2, and the total number of cells and the ratio of ACTA2+ALDH1A2- SMCs, ACTA2-ALDH1A2+ LP fibrocytes, and ACTA2-ALDH1A2- "undifferentiated" LP cells were determined. (**B**) Cultures were additionally stained for the SMC marker ACTA2 and the urothelial marker CDH1 and the total number of cells and the ratio of ACTA2+CDH1- SMCs and ACTA2-CDH1- LP cells were determined.

Available for download at
https://journals.biologists.com/dev/article-lookup/doi/10.1242/dev.204214#supplementary-data

Table S4. Effect of increasing concentrations of IWP-2 on lamina propria cells in cultures of E12.5 ureters (A relates to Fig. 3B and B to Figure S6B). Ureters were explanted at E12.5 and cultured for 2 days without IWP-2 and for a further 6 days with increasing concentrations of IWP-2. (**A**) Cultures were then processed for expression of the smooth muscle cell (SMC) marker ACTA2 and the lamina propria (LP) marker ALDH1A2, and the total number of cells and the ratio of ACTA2+ALDH1A2- SMCs, ACTA2-ALDH1A2+ LP fibrocytes, and ACTA2-ALDH1A2- "undifferentiated" LP cells were determined. (**B**) Cultures were additionally stained for the SMC marker ACTA2 and the urothelial marker CDH1 and the total number of cells and the ratio of ACTA2+CDH1- SMCs and ACTA2-CDH1- LP cells were determined.

Available for download at
https://journals.biologists.com/dev/article-lookup/doi/10.1242/dev.204214#supplementary-data

Table S5. Effect of increasing concentrations of BIO on lamina propria cells in cultures of E12.5 ureters (A relates to Fig. 3B and B to Fig. S6B). Ureters were explanted at E12.5 and cultured for 2 days without BIO and for a further 6 days with increasing concentrations of BIO. (**A**) Cultures were then processed for expression of the smooth muscle cell (SMC) marker ACTA2 and the lamina propria (LP) marker ALDH1A2, and the total number of cells and the ratio of ACTA2+ALDH1A2- SMCs, ACTA2-ALDH1A2+ LP fibrocytes, and ACTA2-ALDH1A2- “undifferentiated” LP cells were determined. (**B**) Cultures were additionally stained for the SMC marker ACTA2 and the urothelial marker CDH1 and the total number of cells and the ratio of ACTA2+CDH1- SMCs and ACTA2-CDH1- LP cells were determined.

Available for download at
https://journals.biologists.com/dev/article-lookup/doi/10.1242/dev.204214#supplementary-data

Table S6. Effect of increasing concentrations of NOG on lamina propria cells in cultures of E12.5 ureters (A relates to Fig. 3C and B to Fig. S6C). Ureters were explanted at E12.5 and cultured for 2 days without NOG and for a further 6 days with increasing concentrations of NOG. (**A**) Cultures were then processed for expression of the smooth muscle cell (SMC) marker ACTA2 and the lamina propria (LP) marker ALDH1A2, and the total number of cells and the ratio of ACTA2+ALDH1A2- SMCs, ACTA2-ALDH1A2+ LP fibrocytes, and ACTA2-ALDH1A2- “undifferentiated” LP cells were determined. (**B**) Cultures were additionally stained for the SMC marker ACTA2 and the urothelial marker CDH1 and the total number of cells and the ratio of ACTA2+CDH1- SMCs and ACTA2-CDH1- LP cells were determined.

Available for download at
https://journals.biologists.com/dev/article-lookup/doi/10.1242/dev.204214#supplementary-data

Development • Supplementary information

Table S7. Effect of increasing concentrations of BMP4 on lamina propria cells in cultures of E12.5 ureters (A relates to Fig. 3C and B to Fig. S6C). Ureters were explanted at E12.5 and cultured for 2 ys without BMP4 and for a further 6 days with increasing concentrations of BMP4. (**A**) Cultures were then processed for expression of the smooth muscle cell (SMC) marker ACTA2 and the lamina propria (LP) marker ALDH1A2, and the total number of cells and the ratio of ACTA2+ALDH1A2- SMCs, ACTA2-ALDH1A2+ LP fibrocytes, and ACTA2-ALDH1A2- “undifferentiated” LP cells were determined. (**B**) Cultures were additionally stained for the SMC marker ACTA2 and the urothelial marker CDH1 and the total number of cells and the ratio of ACTA2+CDH1- SMCs and ACTA2-CDH1- LP cells were determined.

Available for download at
https://journals.biologists.com/dev/article-lookup/doi/10.1242/dev.204214#supplementary-data

Table S8. Effect of increasing concentrations of BMS493 on lamina propria cells in cultures of E12.5 ureters (A relates to Fig. 3D and B to Fig. S6D). Ureters were explanted at E12.5 and cultured for 2 days without BMS493 and for a further 6 days with increasing concentrations of BMS493. (**A**) Cultures were then processed for expression of the smooth muscle cell (SMC) marker ACTA2 and the lamina propria (LP) marker ALDH1A2, and the total number of cells and the ratio of ACTA2+ALDH1A2- SMCs, ACTA2-ALDH1A2+ LP fibrocytes, and ACTA2-ALDH1A2- “undifferentiated” LP cells were determined. (**B**) Cultures were additionally stained for the SMC marker ACTA2 and the urothelial marker CDH1 and the total number of cells and the ratio of ACTA2+CDH1- SMCs and ACTA2-CDH1- LP cells were determined.

Available for download at
https://journals.biologists.com/dev/article-lookup/doi/10.1242/dev.204214#supplementary-data

Table S9. Effect of increasing concentrations of retinoic acid (RA) on lamina propria cells in cultures of E12.5 ureters (A relates to Fig. 3D and B to Fig. S6D). Ureters were explanted at E12.5 and cultured for 2 days without retinoic acid (RA) and for a furth 6 days with increasing concentrations of RA. (**A**) Cultures were then processed for expression of the smooth muscle cell (SMC) marker ACTA2 and the lamina propria (LP) marker ALDH1A2, and the total number of cells and the ratio of ACTA2+ALDH1A2- SMCs, ACTA2-ALDH1A2+ LP fibrocytes, and ACTA2-ALDH1A2- “undifferentiated” LP cells were determined. (**B**) Cultures were additionally stained for the SMC marker ACTA2 and the urothelial marker CDH1 and the total number of cells and the ratio of ACTA2+CDH1- SMCs and ACTA2-CDH1- LP cells were determined.

Available for download at
https://journals.biologists.com/dev/article-lookup/doi/10.1242/dev.204214#supplementary-data

Table S10. Effect of increasing concentrations of cyclopamine on lamina propria cells in cultures of P0 ureters (A relates to Fig. 4A and B to Fig. S7A). Ureters were explanted at P0 and cultured for 6 days with increasing concentrations of cyclopamine. (**A**) Cultures were then processed for expression of the smooth muscle cell (SMC) marker ACTA2 and the lamina propria (LP) marker ALDH1A2, and the total number of cells and the ratio of ACTA2+ALDH1A2- SMCs, ACTA2-ALDH1A2+ LP fibrocytes, and ACTA2-ALDH1A2- “undifferentiated” LP cells were determined. (**B**) Cultures were additionally stained for the SMC marker ACTA2 and the urothelial marker CDH1 and the total number of cells and the ratio of ACTA2+CDH1- SMCs and ACTA2-CDH1- LP cells were determined.

Available for download at
https://journals.biologists.com/dev/article-lookup/doi/10.1242/dev.204214#supplementary-data

Table S11. Effect of increasing concentrations of purmorphamine on lamina propria cells in cultures of P0 ureters (A relates to Fig. 4A and B to Fig. S7A). Ureters were explanted at P0 and cultured for 6 days with increasing concentrations of purmorphamine. (**A**) Cultures were then processed for expression of the smooth muscle cell (SMC) marker ACTA2 and the lamina propria (LP) marker ALDH1A2, and the total number of cells and the ratio of ACTA2+ALDH1A2- SMCs, ACTA2-ALDH1A2+ LP fibrocytes, and ACTA2-ALDH1A2- “undifferentiated” LP cells were determined. (**B**) Cultures were additionally stained for the SMC marker ACTA2 and the urothelial marker CDH1 and the total number of cells and the ratio of ACTA2+CDH1- SMCs and ACTA2-CDH1- LP cells were determined.

Available for download at
https://journals.biologists.com/dev/article-lookup/doi/10.1242/dev.204214#supplementary-data

Table S12. Effect of increasing concentrations of IWP-2 on lamina propria cells in cultures of P0 ureters (A relates to Fig. 4B and B to Fig. S7B). Ureters were explanted at P0 and cultured for 6 days with increasing concentrations of IWP-2. (**A**) Cultures were then processed for expression of the smooth muscle cell (SMC) marker ACTA2 and the lamina propria (LP) marker ALDH1A2, and the total number of cell and the ratio of ACTA2 +ALDH1A2- SMCs, ACTA2-ALDH1A2+ LP fibrocytes, and ACTA2-ALDH1A2- “undifferentiated” LP cells were determined. (**B**) Cultures were additionally stained for the SMC marker ACTA2 and the urothelial marker CDH1 and the total number of cells and the ratio of ACTA2+CDH1- SMCs and ACTA2-CDH1- LP cells were determined.

Available for download at
https://journals.biologists.com/dev/article-lookup/doi/10.1242/dev.204214#supplementary-data

Development • Supplementary information

Table S13. Effect of increasing concentrations of BIO on lamina propria cells in cultures of P0 ureters (A relates to Fig. 4B and B to Fig. S7B). Ureters were explanted at P0 and cultured for 6 days with increasing concentrations of BIO. (**A**) Cultures were then processed for expression of the smooth muscle cell (SMC) marker ACTA2 and the lamina propria (LP) marker ALDH1A2, and the total number of cells and the ratio of ACTA2+ALDH1A2- SMCs, ACTA2-ALDH1A2+ LP fibrocytes, and ACTA2-ALDH1A2- "undifferentiated" LP cells were determined. (**B**) Cultures were additionally stained for the SMC marker ACTA2 and the urothelial marker CDH1 and the total number of cells and the ratio of ACTA2+CDH1- SMCs and ACTA2-CDH1- LP cells were determined.

Available for download at
https://journals.biologists.com/dev/article-lookup/doi/10.1242/dev.204214#supplementary-data

Table S14. Effect of increasing concentrations of NOG on lamina propria cells in cultures of P0 ureters (A relates to Fig. 4C and B to Fig. S7C). Ureters were explanted at P0 and cultured for 6 days with increasing concentrations of NOG. (**A**) Cultures were then processed for expression of the smooth muscle cell (SMC) marker ACTA2 and the lamina propria (LP) marker ALDH1A2, and the total number of cells and the ratio of ACTA2+ALDH1A2- SMCs, ACTA2-ALDH1A2+ LP fibrocytes, and ACTA2-ALDH1A2- "undifferentiated" LP cells were determined. (**B**) Cultures were additionally stained for the SMC marker ACTA2 and the urothelial marker CDH1 and the total number of cells and the ratio of ACTA2+CDH1- SMCs and ACTA2-CDH1- LP cells were determined.

Available for download at
https://journals.biologists.com/dev/article-lookup/doi/10.1242/dev.204214#supplementary-data

Table S15. Effect of increasing concentrations of BMP4 on lamina propria cells in cultures of P0 ureters (A relates to Fig. 4C and B to Fig. S7C). Ureters were explanted at P0 and cultured for 6 days with increasing concentrations of BMP4. (**A**) Cultures were then processed for expression of the smooth muscle cell (SMC) marker ACTA2 and the lamina propria (LP) marker ALDH1A2, and the total number of cells and the ratio of ACTA2+ALDH1A2- SMCs, ACTA2-ALDH1A2+ LP fibrocytes, and ACTA2-ALDH1A2- “undifferentiated” LP cells were determined. (**B**) Cultures were additionally stained for the SMC marker ACTA2 and the urothelial marker CDH1 and the total number of cells and the ratio of ACTA2+CDH1- SMCs and ACTA2-CDH1- LP cells were determined.

Available for download at
https://journals.biologists.com/dev/article-lookup/doi/10.1242/dev.204214#supplementary-data

Table S16. Effect of increasing concentrations of BMS493 on lamina propria cells in cultures of P0 ureters (A relates to Figure 4D and B to Figure S7D). Ureters were explanted at P0 and cultured for 6 days with increasing concentrations of BMS493. (**A**) Cultures were then processed for expression of the smooth muscle cell (SMC) marker ACTA2 and the lamina propria (LP) marker ALDH1A2, and the total number of cells and the ratio) of ACTA2+ALDH1A2- SMCs, ACTA2-ALDH1A2+ LP fibrocytes, and ACTA2-ALDH1A2- “undifferentiated” LP cells were determined. (**B**) Cultures were additionally stained for the SMC marker ACTA2 and the urothelial marker CDH1 and the total number of cells and the ratio of ACTA2+CDH1- SMCs and ACTA2-CDH1- LP cells were determined.

Available for download at
https://journals.biologists.com/dev/article-lookup/doi/10.1242/dev.204214#supplementary-data

Development • Supplementary information

Table S17. Effect of increasing concentrations of retinoic acid (RA) on on lamina propria cells in cultures of P0 ureters (A relates to Figure 4D and B to Figure S7D). Ureters were explanted at P0 and cultured for 6 days with increasing concentrations of retinoic acid (RA). (**A**) Cultures were then processed for expression of the smooth muscle cell (SMC) marker ACTA2 and the lamina propria (LP) marker ALDH1A2, and the total number of cells (nd the ratio (ACTA2+ALDH1A2- SMCs, ACTA2-ALDH1A2+ LP fibrocytes, and ACTA2-ALDH1A2- “undifferentiated” LP cells were determined. (**B**) Cultures were additionally stained for the SMC marker ACTA2 and the urothelial marker CDH1 and the total number of cells and the ratio of ACTA2+CDH1- SMCs and ACTA2-CDH1- LP cells were determined.

Available for download at
https://journals.biologists.com/dev/article-lookup/doi/10.1242/dev.204214#supplementary-data

Table S18. Effect of combination of SHH and WNT activators and inhibitors on lamina propria cells in cultures of P0 ureters (A relates to Fig. 5B and B relates to Fig. 5C). Ureters were explanted at P0 and cultured for 6 days with different combinations of SHH and WNT activators (purmorphamine and BIO) and inhibitors (cyclopamine and IWP-2). (**A**) Cultures were then processed for expression of the smooth muscle cell (SMC) marker ACTA2 and the lamina propria (LP) marker ALDH1A2, and the total number of cells and the ratio of ACTA2+ALDH1A2- SMCs, ACTA2-ALDH1A2+ LP fibrocytes, and ACTA2-ALDH1A2- “undifferentiated” LP cells were determined. (**B**) Cultures were additionally stained for the SMC marker ACTA2 and the urothelial marker CDH1 and the total number of cells and the ratio of ACTA2+CDH1- SMCs and ACTA2-CDH1- LP cells were determined.

Available for download at
https://journals.biologists.com/dev/article-lookup/doi/10.1242/dev.204214#supplementary-data

Table S19. Effect of combination of SHH activation and BMP4 inhibition on lamina propria cells in cultures of E12.5, E12.5+2d and P0 ureters (relates to Figure 5E). Ureters were explanted at E12.5 and P0. The E12.5 ureters were cultured for 2 days without and then cultured for 6 days with SHH activator purmorphamine and the BMP4 inhibitor NOG. P0 ureters were directly cultured with SHH activator purmorphamine and the BMP4 inhibitor NOGGIN. (**A**) Cultures were then processed for expression of the smooth muscle cell (SMC) marker ACTA2 and the lamina propria (LP) marker ALDH1A2, and the total number of cells and the ratio of ACTA2+ALDH1A2- SMCs, ACTA2-ALDH1A2+ LP fibrocytes, and ACTA2-ALDH1A2- "undifferentiated" LP cells were determined.

Available for download at
https://journals.biologists.com/dev/article-lookup/doi/10.1242/dev.204214#supplementary-data

Table S20. Statistical analysis of mesenchymal cell proliferation in 18 h cultures of P0 ureter explants treated with individual signaling pathway inhibitors and BrdU (relates to Fig. 6C and 6D). After the 18 hours culture period ureters were fixed, embedded in paraffine-wax and sectioned to 5-µm before performing co-immunofluorescence analysis for ACTA2 and CDH1 expression and BrdU insertion. The total number of ACTA2+ smooth muscle cells (SMCs) and ACTA2- CDH1- lamina propria (LP) cells was determined, as was the ratio of BrdU+ ACTA2+ CDH- SMCs and BrdU+ ACTA2- CDH- LP fibrocytes.

Available for download at
https://journals.biologists.com/dev/article-lookup/doi/10.1242/dev.204214#supplementary-data

Development • Supplementary information

Table S21. List of genes with decreased expression in micoroarrays of explants of P0 ureters treated for 18 h with 10 µM cyclopamine (relates to Fig. 7A and 7C). Shown are the gene names, the intensity of the two control and mutant ureter samples, the individual and the average (avg) fold change (FC). Genes which are also dowregulated in P0 ureters treated with 5 µ M IWP-2 are marked in red (significant in all pools), and in green (significant in one of the two pools).

Available for download at
https://journals.biologists.com/dev/article-lookup/doi/10.1242/dev.204214#supplementary-data

Table S22. Functional annotation by DAVID for genes with decreased expression in P0 ureters treated for 18 h with 10 µM of the SHH signaling inhibitor cyclopamine (relates to Fig. 7B).

Available for download at
https://journals.biologists.com/dev/article-lookup/doi/10.1242/dev.204214#supplementary-data

Table S23. List of genes with decreased expression in micoroarrays of explants of P0 ureters treated for 18 h with 5 µM IWP-2 (relates to Fig. 7D and 7F). Shown are the gene names, the intensity of the two control and mutant ureter samples, the individual and the average (avg) fold change (FC). Genes which are also dowregulated in P0 ureters treated with 10 µ M cyclopamine are marked in red (significant in all pools), and in green (significant in one of the two pools).

Available for download at
https://journals.biologists.com/dev/article-lookup/doi/10.1242/dev.204214#supplementary-data

Development • Supplementary information

Table S24. Functional annotation by DAVID for genes with decreased expression in P0 ureters treated for 18 h with 5 µ M of the WNT signaling inhibitor IWP-2 (relates to Fig. 7E).

Available for download at
https://journals.biologists.com/dev/article-lookup/doi/10.1242/dev.204214#supplementary-data

Table S25. List of antibodies for immunofluoresent detection of antigens on paraffin wax sections (relates to materials and methods).

Available for download at
https://journals.biologists.com/dev/article-lookup/doi/10.1242/dev.204214#supplementary-data
