# Supplementary material for: Interplay of SHH, WNT and BMP4 signaling regulates the development of the lamina propria in the murine ureter
Source: Development. 2025 Feb 6;152(3):DEV204214. doi: 10.1242/dev.204214 (PMC11829765; doi:10.1242/dev.204214)
Supplement: Supplementary file 1 [file develop-152-204214_review_history.pdf]

# Interplay of SHH, WNT and BMP4 signaling regulates the development of the lamina propria in the murine ureter

Philipp Straube, Anja Beckers, Ulrich Jany, Florian Bergmann, Timo Lüdtke, Carsten Rudat, Mark-Oliver Trowe, Imke Peters, Maximilian G. Klopf, Tamrat Mamo, Andreas Kispert
DOI: 10.1242/dev.204214

**Editor**: Liz Robertson

**Review timeline**

| | |
|---|---|
| Original submission: | 27 June 2024 |
| Editorial decision: | 20 August 2024 |
| First revision received: | 12 December 2024 |
| Accepted: | 31 December 2024 |

---

**Original submission**

First decision letter

MS ID#: dev.204214

MS TITLE: Interplay of SHH, WNT and BMP4 signaling regulates the development of the lamina propria in the murine ureter

AUTHORS: Philipp Straube, Anja Beckers, Ulrich Jany, Florian Bergmann, Timo Lüdtke, Carsten Rudat, Mark-Oliver Trowe, Imke Peters, Maximilian G. Klopf, Tamrat Mamo and Andreas Kispert

I have now received all the referees reports on the above manuscript, and have reached a decision. The referees' comments are appended below, or you can access them online: please go to.

The overall evaluation is positive and we would like to publish a revised manuscript in Development, provided that the referees' comments can be satisfactorily addressed. Please attend to all of the reviewers' comments in your revised manuscript and detail them in your point-by-point response. If you do not agree with any of their criticisms or suggestions explain clearly why this is so. If it would be helpful, you are welcome to contact us to discuss your revision in greater detail. Please send us a point-by-point response indicating your plans for addressing the referees' comments, and we will look over this and provide further guidance.

Reviewer 1

*Advance summary and potential significance to field*

In this manuscript Straube et al., investigate the signaling mechanisms that regulate the development of the ureteral lamina propria in a mouse ureter. They focus on 4 major signaling pathways (SHH, WNT, BMP and retinoic acid (RA)) that have previously been demonstrated to play critical roles in ureter development. Using embryonic ureter organ cultures combined with small molecule agonists and antagonists of the mentioned signaling pathways and staining for ureteral lineage markers the authors compellingly demonstrate that the development of lamina propria is controlled by SHH and WNT signaling activity. In contrast, inhibiting or enhancing BMP4 or RA activity does not significantly affect lamina propria specification in the developing ureter. However, the authors demonstrate that, unexpectedly, BMP4 plays a key instructive role for the

© 2025. Published by The Company of Biologists under the terms of the Creative Commons Attribution License (https://creativecommons.org/licenses/by/4.0/).

smooth muscle cell lineage formation during early stages (around E12.5) of ureter morphogenesis - inhibition of BMP signaling by NOGGIN abolishes the smooth muscle fate at the expense of lamina propria fibrocyte formation. This new finding provides an important detail to the regulation of early ureter development. The experimental findings in ureter explant cultures are supported by unbiased microarray analysis of wild type, cyclopamine (SHHi) or IWP-2 (WNTi) treated ureters. The manuscript is very well written, experiments are logical and the results highly impactful in the field of urinary system development. Phenotypic analyses are of high quality and the in vitro mechanistic studies using signaling pathway modulators carefully performed. All studies are well powered.

*Comments for the author*

The reviewer has no major comments.

Minor comments.

Line 94-95. Please rephrase the second part of the sentence at the end of the Introduction "…, lack of BMP4 signaling rules the LP fate", for clarity.

Figure 2. Axin2 in situ figure on day P7 is shifted.

Reviewer 2

*Advance summary and potential significance to field*

While much is known regarding the patterning and specification of bladder and ureteral urothelium and smooth muscle, relatively little is known pertaining to the development and maintenance of the ureteral lamina propria (LP). Here, Straube et al investigate the development of the ureteral LP during embryonic and postnatal stages. The authors used immunofluorescent localization to determine the onset of the LP fate, and profiled SHH, WNT, BMP4, and RA signaling using RNA in situ hybridization, and used microarray analysis to analyze the impact of SHH and WNT signaling inhibition. Using ureteral explants and pharmacological manipulation, Straube et al investigated the roles of these pathways in the development and maintenance of the LP. Briefly, they defined ALDH1A2 as a marker for ureteral LP, and discovered that SHH and WNT signaling promote LP and smooth muscle cell proliferation and differentiation in the ureter, and present a role for BMP4 in diversifying the mesenchymal compartment into LP and SMCs. This lab is recognized as world class experts in ureteral development, and the data presented are exhaustive. This reviewer has outlined critiques aimed at improving, and in some cases simplifying, this manuscript for a broad developmental biology audience.

*Comments for the author*

Line 63-67. "We have recently shown by genetic lineage tracing…" (awkward use of the word 'recent' to describe a 2013 publication.

Figure 1
* Line 111-112. "At E16.5, the cells of the inner UM expressed ACTA2, indicating SMC differentiation." ACTA2 can be expressed by SMCs and activated fibroblasts; therefore, it alone cannot indicate SMC differentiation.
* Line 114-120. Authors conclude an a major expansion of the LP occurs after birth by an increased number of fibrocytes without quantification.

Figure 2
* Why is P40 not included? This is especially important given the authors reported dramatic difference in collagen and ALDH1A2 at P7 - P40.
* In several images, it is difficult to discern UE vs UM or LP and TM and distinguish "B" cells of the UE. Co-detection of tissue specific proteins (ie ALDH1A2, TAGLN, KRT5) and probe would clarify (RNAscope co-detection).

© 2025. Published by The Company of Biologists under the terms of the Creative Commons Attribution License (https://creativecommons.org/licenses/by/4.0/). 

* Line 129. "Shh was expressed at low levels in the UE at E14.5 and E16.5." This is difficult to discern in the absence of a negative control.
* Line 131/142. Ptch is introduced as a target gene of SHH signaling, and Rarb is introduced as a direct target gene of RA signaling. Also introducing these as receptors for SHH and RA, may make it easier to understand how the 'target' is highly expressed in the absence or low expression of SHH and RA. This is especially the case for E14.5 & E16.5. Other regulators may be needed to support the rationale that these pathways are active.
* Line 133. Authors claim Wnt7b resembles Shh expression pattern. While both are expressed by the urothelium, it appears that Shh is higher at E18.5-P7, while Wnt7b is higher at E14.5.
* Line 140-141: "Aldh1a2, was weakly expressed in the UM at E14.5." It is difficult to appreciate weak expression of Aldh1a2 in the UM at this stage. This is especially important since Aldh1a2 is apparently not expressed at E16.6 and then (re-)occurred in LP at ≥E18.5.

Figure S2/S3
* It is unclear from the text why the ureter + kidney were isolated en bloc for E12.5 explant studies. Furthermore, RT-qPCR would provide a more sensitive and WB more conclusive assessment of pathway activation/inhibition for both explant time points.

Figure 3
* Line 177. Would be key to show that E12.5 + 2 days indeed exhibit expression patterns expected from E13.5-E14.0 in vivo. Also, could this experiment be done using E14.5 ureters (without kidney) in a manner that does not require the 2 day media-only incubation period?
* Line 186-188. Authors mentioned that "BIO treatment led to a dose-dependent increase of the overall cell number largely due to gain of "undifferentiated" LP cells." Puromorphamine treatment also led to a dose-dependent increase of the overall cell number (though not due to a gain of undifferentiated cells, but rather LP cells), yet the assessment of puromorphamine was described only as "had the opposite effect" of cyclopamine, on Line 184-185.

Figure 4.
* Line 213-215. "Purmorphamine led to a reciprocal dose-dependent increase of both SMCs and LP fibrocytes but SMCs were disproportionally increased." Consider indicating that the SMCs were disproportionately increased at 10uM.
* Line 215-216. "Inhibition of WNT signaling by IWP-2 led to a dose-dependent equal reduction of LP fibrocytes and SMCs." SMCs; however, do not appear to be affected by IWP-2 treatment.
* Line 221-223. BMP4 treatment significantly impacts LP and SMC ratio at the two highest doses, but is not mentioned in the summary of findings. Instead, WNT signaling is mentioned even though WNT signaling does not affect the ratio of LP and SMCs at any dose. Furthermore, it would be simple to state that these findings suggest that SHH signaling is required for maintenance of ALDH1A2+ LP cells (rather than state "These findings suggest that SHH signaling is required for ALDH1A2 expression, hence differentiation of LP fibrocytes," because this was a maintenance stage experiment.

Figure 5.
* "Combined treatment with cyclopamine and IWP-2 led to an almost complete loss of ALDH1A2+ LP fibrocytes and a strong reduction of ACTA2+ SMCs, enhancing the effects of individual pathway inhibition." It is difficult to appreciate how combined treatment ENHANCED the effects of individual pathway inhibition given that cyclopamine treatment alone at 5uM led to an average of 0 LP cells, and at 10uM led to an average of 1 LP cell (Fig 4A, Table S9).
* Consider re-ordering the data in Fig. 5 to align with how it is introduced in the main text (ie. 1) Control, 2) IWP-2/Cyclo, 3) BIO/Puro, 4) IWP-2/Puro, 5) Cyclo/BIO).
* There appears to be a typo in labeled concentration of Puromorph. in Fig 5. panel A (10uM v 2uM). The image shows 10uM, but the graph reports 2uM. If 2uM was used, why was that concentration of puro chosen when 10uM, but not 2uM showed a significant increase in LP AND SMCs in Fig. 4?
* Line. 235. "WNT signaling activation rescued LP cell number but not ALDH1A2 expression when SHH signaling was inhibited." Consider revising: WNT signaling activation did not rescue ALDH1A2+ LP cell number when SHH signaling was inhibited, but did expand the number of undifferentiated LP cells.

© 2025. Published by The Company of Biologists under the terms of the Creative Commons Attribution License (https://creativecommons.org/licenses/by/4.0/).

* Line 237-238. "We conclude that SHH and WNT signaling act in parallel in postnatal LP development." In my opinion, the data suggest that WNT promotes expansion of undifferentiated LP cells, while SHH promotes LP differentiation.
* Line 225-226. A little confusing to title this results section "LP development," when previously using E12.5 explants for LP development and P0 explants for LP maintenance studies. Would E12.5 explants be better suited for the SHH/WNT study?
* In my opinion, pseudocolor the IF markers: ACTA2 (green), and ALDH1A2 (red) to match the 1) bar colors for the graphs in Fig. 5 panel E, 2) images in Fig. 5 panel A, and 3) Figures S4 and S5.
* Line 243-244. "In E14.5 and E18.5 explants, normal patterning into LP cells and SMCs occurred." There graphs show statistically significant changes in ALDH1A2+ LP cells and ACTA2+ SMCs, which makes it difficult to appreciate normal patterning occurred. Furthermore, the expression of ACTA2 is reduced (would require WB or qPCR to assess expression levels) and the SMCs appear disorganized, which suggests BMP4 is important for 'maintaining' the SMC phenotype.
* "Ectopic SHH signaling and ALDH1A2 expression cannot overwrite the SMC phenotype." Awkward summary statement considering this experiment did not simply address ectopic SHH signaling.

Figure 6.
* Line 254. Why were E18.5 explants used for this study which is aimed at addressing "the observed changes in cell numbers" and why were signaling inhibitors used for 48h for this study? Since the entire panel of inhibitors is used and the most prominent changes in cell number occurred in Fig. 3, this experiment would make more sense if modeled in the same way and the data would flow better if presented immediately following that figure. E12.5 LP is as difficult to discern as the E18.5 LP.
* I can't find where the definition of the annotation is panel A is defined, but the elliptical annotation is not an authentic representation of the basement membrane. However, this type of annotation that demarcates the space between the TM and UE basement membrane would be helpful, here and elsewhere.

Figure 7.
* Line 266. Why change IWP-2 to 10uM when 5uM has been used previously in E18 explant?
* Line 275. Clarify 104 downregulated DEGs.
* Unclear why the microarray is E18.5 + 18h treatment, while in situ hybridization validation is E14.5, P0, and P0 + 18h explants.
* Consider changing the format of Fig. 3 and Fig. S4, and Fig. 4 and Fig. S5. It would be helpful to include the left side image panels from Fig. S4 next to the left side graphs from Fig. 3 for the E12.5 explants. Likewise, it would be helpful to include the left side image panels from Fig. S5 next to the left side graphs from Fig 4 for the P0 explants. The main text makes little mention of the CDH1 ureteral urothelium data, so the Figs. 3/4 right side graphs may fit better in the data supplement with the images pulled from the right sides of Fig. S4/S5.
* Figure legends report "two-tailed Student's t-test, Welch's t-test or Mann-Whitney U test" - which statistical test is indicated on the graphs?

Discussion
* Comments outlined for each Figure point to criticisms that limit the impact of some of the points made by the authors in the discussion. Overall, the discussion section of this manuscript is not nearly as exhaustive as the data presented in this manuscript. A careful discussion of the data presented in this manuscript would require more attention to 1) development vs maintenance phases examined, 2) attention to the order in which topics are presented, and 3) a careful comparison of the findings and those in the literature, including important technical details and contexts.
* Line 301. LP is referred to as developing into a "thick cell layer" at early postnatal stages. In my opinion, the ureteral LP does not appear thick at either P0 or P7 - perhaps thickness measurements would support the opportunity to say there is a statistically significant increase in thickness. Otherwise, I suggest dropping the word thick, and referring to the emergence of a morphologically distinct LP.
* Claims that inhibition of SHH and WNT signaling abolished the LP do not seem appropriate when undifferentiated LP cells persist. Instead, inhibition of SHH and WNT signaling led to a dramatic loss of ALDH1A2, which represents a state of differentiation for the LP cells.
* Line 329-335. This is a cumbersome sentence.

© 2025. Published by The Company of Biologists under the terms of the Creative Commons Attribution License (https://creativecommons.org/licenses/by/4.0/).

* Line 326. Should this say: In explants of E12.5/P0 ureters?
* Final sentence of the discussion ("…contribute to fibrotic programs that occur in the ureter during injury or obstruction") seems random without a little more effort to integrate this into the discussion.

Methods.
* Please mention the software and methods used to quantitate positive and negative cell counts. This is important considering that with the images presented, the boundaries of the cell types (especially when the manipulation impacts the LP and UE).
* Define the conditions for the 'controls' for the combination experiments (Ethanol/DMSO/W etc.).

---

**First revision**

Author response to reviewers' comments

**Response to reviewers**

**Reviewer 1:**

SUMMARY OF THE ADVANCE MADE IN THIS PAPER AND ITS POTENTIAL SIGNIFICANCE TO THE FIELD
In this manuscript Straube et al., investigate the signaling mechanisms that regulate the development of the ureteral lamina propria in a mouse ureter. They focus on 4 major signaling pathways (SHH, WNT, BMP and retinoic acid (RA)) that have previously been demonstrated to play critical roles in ureter development. Using embryonic ureter organ cultures combined with small molecule agonists and antagonists of the mentioned signaling pathways and staining for ureteral lineage markers the authors compellingly demonstrate that the development of lamina propria is controlled by SHH and WNT signaling activity. In contrast, inhibiting or enhancing BMP4 or RA activity does not significantly affect lamina propria specification in the developing ureter. However, the authors demonstrate that, unexpectedly, BMP4 plays a key instructive role for the smooth muscle cell lineage formation during early stages (around E12.5) of ureter morphogenesis - inhibition of BMP signaling by NOGGIN abolishes the smooth muscle fate at the expense of lamina propria fibrocyte formation. This new finding provides an important detail to the regulation of early ureter development. The experimental findings in ureter explant cultures are supported by unbiased microarray analysis of wild type, cyclopamine (SHHi) or IWP-2 (WNTi) treated ureters. The manuscript is very well written, experiments are logical and the results highly impactful in the field of urinary system development. Phenotypic analyses are of high quality and the in vitro mechanistic studies using signaling pathway modulators carefully performed. All studies are well powered.

>> We are grateful for the very positive words of this reviewer for the quality and impact of our manuscript. <<

SUGGESTIONS TO AUTHORS
The reviewer has no major comments.
Minor comments.

Line 94-95. Please rephrase the second part of the sentence at the end of the Introduction "…, lack of BMP4 signaling rules the LP fate", for clarity.

>> We have rephrased the second part of the sentence to „, it is the lack of BMP4 signaling, which favors the LP over the SMC fate.“ <<

Figure 2. Axin2 in situ figure on day P7 is shifted.

>> We have corrected this in revised Figure 2. <<

© 2025. Published by The Company of Biologists under the terms of the Creative Commons Attribution License (https://creativecommons.org/licenses/by/4.0/).

**Reviewer 2:**

SUMMARY OF THE ADVANCE MADE IN THIS PAPER AND ITS POTENTIAL SIGNIFICANCE TO THE FIELD
While much is known regarding the patterning and specification of bladder and ureteral urothelium and smooth muscle, relatively little is known pertaining to the development and maintenance of the ureteral lamina propria (LP). Here, Straube et al investigate the development of the ureteral LP during embryonic and postnatal stages. The authors used immunofluorescent localization to determine the onset of the LP fate, and profiled SHH, WNT, BMP4, and RA signaling using RNA in situ hybridization, and used microarray analysis to analyze the impact of SHH and WNT signaling inhibition. Using ureteral explants and pharmacological manipulation, Straube et al investigated the roles of these pathways in the development and maintenance of the LP. Briefly, they defined ALDH1A2 as a marker for ureteral LP, and discovered that SHH and WNT signaling promote LP and smooth muscle cell proliferation and differentiation in the ureter, and present a role for BMP4 in diversifying the mesenchymal compartment into LP and SMCs. This lab is recognized as world class experts in ureteral development, and the data presented are exhaustive. This reviewer has outlined critiques aimed at improving, and in some cases simplifying, this manuscript for a broad developmental biology audience.

>> We would like to thank the reviewer for taking the time to carefully read and evaluate our manuscript. We are very happy about the strong words of appreciation on the quality and relevance of our work. <<

QUESTIONS TO AUTHORS

Line 63-67. "We have recently shown by genetic lineage tracing…" (awkward use of the word 'recent' to describe a 2013 publication.

>> We have rephrased this to: Genetic lineage tracing revealed that in the developing ureter of the mouse,…” <<

Figure 1.

* Line 111-112. "At E16.5, the cells of the inner UM expressed ACTA2, indicating SMC differentiation." ACTA2 can be expressed by SMCs and activated fibroblasts; therefore, it alone cannot indicate SMC differentiation.

>> The reviewer is right. ACTA2 on its own is found both in SMCs and myofibroblasts. To more carefully define the SMC fate, we now additionally use TAGLN, CKM and SMTN as markers (new Fig. S1).

In the result section (p6, first paragraph), we now write: “Combinatorial cytoplasmic expression of ACTA2 and TAGLN identified SMCs of an early differentiation state; a late stage of differentiation was characterized by additional expression of CKM and SMTN in the cytoplasm of these cells”.

In the second paragraph, we write: “SMC markers and ALDH1A2 were not expressed. At E16.5, the cells of the inner UM co-expressed ACTA2 and TAGLN, indicating early SMC differentiation. At E18.5 and postnatal day (P)0, patches of ALDH1A2$^+$ cells were found in between the ACTA2$^+$TAGLN$^+$CKM$^+$SMTN$^+$ late SMCs and the CDH1$^+$ urothelium. At P7 and P40, the population of SMC marker-negative mesenchymal cells underneath the urothelium appeared largely expanded, and expressed ALDH1A2 homogenously.” <<

* Line 114-120. Authors conclude a major expansion of the LP occurs after birth by an increased number of fibrocytes without quantification.

>> We thank for the advice to strengthen our conclusion by an additional quantification of

© 2025. Published by The Company of Biologists under the terms of the Creative Commons Attribution License (https://creativecommons.org/licenses/by/4.0/).

the LP cells. We now show in new Fig.1E that the number of LP fibrocytes increases strongly between P0 and P7.
Moreover, we show in new Fig. 1F that the thickness of the LP layer increases in this time interval but also subsequently (until P40). We accordingly write at the end of the last paragraph of this section:
“The major expansion of the LP occurs after birth and is associated with a strong increase of LP fibrocytes until P7, and collagen deposition until and after this stage.”
<<

Figure 2
* Why is P40 not included? This is especially important given the authors reported dramatic difference in collagen and ALDH1A2 at P7 - P40.

>> We tried several times to perform RNA in situ hybridizations on P40 ureters. We used different fixation protocols to maintain the mRNA integrity in the ureteral tissues. Although we were able to detect signals for various probes in the urothelium and the SMC layer, we failed to detect signals for genes in the LP, even for those for which we were able to see the corresponding protein (ALDH1A2). We deem that the fixation of mRNA in the LP is particularly difficult since the cells are sparse and embedded in a thick extracellular matrix, which might prevent the penetration of the fixative.
Although we regret this technical insufficiency, we deem that analysis of signaling activities at stage P40 would not add much to our story since we show that the major increase of LP fibrocytes (and matrix) occurs between P0 and P7 - stages we have covered well in our analysis. <<

* In several images, it is difficult to discern UE vs UM or LP and TM and distinguish "B" cells of the UE. Co-detection of tissue specific proteins (ie ALDH1A2, TAGLN, KRT5) and probe would clarify (RNAscope co-detection).

>> We disagree with the reviewer that the UE is difficult to discern from the surrounding mesenchymal cell layers. Upon magnification on the screen it is apparent the UE is clearly demarcated from the UM by the very different shape of the cells.
The LP is discernable as a thin layer of cells underneath the epithelium. Expression of Shh in the urothelium at P7 is clearly absent from the large luminal cells, defining a B- and I-cell restricted pattern.
However, to accommodate the concern of the reviewer, we replaced some of the stainings with new ones (particularly for P0 and P7) to provide a stronger signal and a better resolution. We have more thoroughly labeled our images to point out the expression domains to the reader. Moreover, we are now more careful in describing the expression domains of the genes.
We agree with the reviewer that RNAscope presents a possible means to achieve higher cell-type resolution particularly when doing coexpression analysis. However, the costs of RNA scope is still excessively (and in my opinion) unacceptably) high, and is beyond our budget at this moment. We kindly ask the reviewer to accept this limitation. <<
* Line 129. "Shh was expressed at low levels in the UE at E14.5 and E16.5." This is difficult to discern in the absence of a negative control.

>> We have redone the *Shh* expression analysis at E14.5 and E16.5 and present new images that show a clear Shh signal in the urothelium. The expression is weak compared with other probes but now clearly discernable. Please consider A*ldh1a2* expression at E14.5 and E16.5 as negative control for urothelial (*Shh*) expression. <<

* Line 131/142. Ptch is introduced as a target gene of SHH signaling, and Rarb is introduced as a direct target gene of RA signaling. Also introducing these as receptors for SHH and RA, may make it easier to understand how the 'target' is highly expressed in the absence or low expression of SHH and RA. This is especially the case for E14.5 & E16.5. Other regulators may be needed to support the rationale that these pathways are active.

>> To accommodate the suggestion of the reviewer we now write:
“Expression of *Ptch1*, encoding a receptor for SHH and presenting a target gene of SHH

© 2025. Published by The Company of Biologists under the terms of the Creative Commons Attribution License (https://creativecommons.org/licenses/by/4.0/).

signaling (Ingham and McMahon, 2001), was found in the inner region of the UM at E14.5 and E16.5, and from E18.5 onwards in the LP" and later:
"*Rarb*, encoding a receptor of RA and presenting a direct target gene of RA signaling…
We disagree with the reviewer that we need additional read-outs for pathway activities. The four chosen genes, *Ptch1, Axin2, Id2* and *Rarb* have been established as reliable, suitable and convenient read-outs for the activities of SHH, WNT BMP4 and RA signaling in many studies. And in fact, we show that they respond to pathway inactivation and activation (Fig. S3 and Fig. S4.) <<

* Line 133. Authors claim Wnt7b resembles Shh expression pattern. While both are expressed by the urothelium, it appears that Shh is higher at E18.5-P7, while Wnt7b is higher at E14.5.

>> We are now more careful in our description and write: "*Wnt7b* was strongly expressed in the UE at E14.5 and E16.5; at subsequent stages expression was found at lower levels in the urothelium, most likely in B- and I-cells." <<

* Line 140-141: "Aldh1a2 was weakly expressed in the UM at E14.5." It is difficult to appreciate weak expression of Aldh1a2 in the UM at this stage. This is especially important since Aldh1a2 is apparently not expressed at E16.6 and then (re-)occurred in LP at ≥E18.5.

>> We rechecked expression of *Aldh1a2* at E14.5 and E16.5, and found it to be absent and at background levels, respectively. Therefore, we have changed our description to: "The gene encoding the RA synthesizing enzyme, *Aldh1a2*, was expressed in the LP at E18.5, P0 and P7". <<

Figure S2/S3
* It is unclear from the text why the ureter + kidney were isolated en bloc for E12.5 explant studies.

>> To explain why we grow ureters at E12.5 together with kidneys, we now write: "For this, we explanted ureters together with kidneys at E12.5 (please note that embryonic ureters grow better in this combination culture and stick better on the membrane during later transfer steps) and ureters only at P0 on polyester membranes, cultured them for 18 h at the air-liquid interface in a range of concentrations, and performed whole-mount RNA *in situ* hybridization for direct target genes as a read-out." <<

Furthermore, RT-qPCR would provide a more sensitive and WB more conclusive assessment of pathway activation/inhibition for both explant time points.

>> We agree with the reviewer that RT-PCR would provide a more quantitative and possibly more sensitive approach to check for expression of target genes, hence of pathway activities. However, RT-qPCR analysis, requires at least three pools with many ureters (at E12.5, 15 to 20 each; at P0, 5 each), increasing embryo/animal consumption. Being guided by the 3R rules, we deem that RNA in situ hybridization provides a sufficiently resolved and conclusive estimation of the effects of the various concentrations of the pathway inhibitors and activators, within an acceptable range of animal consumption.
The same argument is valid for Western blots, for which we are also unaware of suitable antibodies for read-outs. <<

Figure 3
* Line 177. Would be key to show that E12.5 + 2 days indeed exhibit expression patterns expected from E13.5-E14.0 in vivo.

>> We changed our words and now write that E12.5 + 2 day ureters should resemble E14.0 to E14.5 ureters. We show that this is indeed the case by analyzing expression of Myocd, ACTA2, TAGLN. We have previously shown that expression of *Myocd* mRNA can be robustly detected at E14.5 while expression of ACTA2 and TAGLN starts at E15.5. In our E12.5+2 day ureters, we found expression of *Myocd* mRNA, while expression of ACTA2 and TAGLN was still absent. This proves that our E12.5 ureter explants reach a stage resembling E14.0 to E14.5 after 2 days in

© 2025. Published by The Company of Biologists under the terms of the Creative Commons Attribution License (https://creativecommons.org/licenses/by/4.0/).

culture.
We show this in new Figure S5. <<

Also, could this experiment be done using E14.5 ureters (without kidney) in a manner that does not require the 2 day media-only incubation period?

>> Yes, this experiment could also be done with E14.5 ureters. We had to switch to E12.5+2 days “pre-cultures” since the legislation was changed in 2023 (in between our experiments) in the federal state of Lower Saxony in Germany. Isolation of fetuses (E13.75 to E19.5) now requires a permit from the authorities. In order to continue the experiments and not to jeopardize the work of the involved PhD students through a lengthy application process for the permit, we switched to the isolation of E12.5 ureters, and the pre-culture system. <<

* Line 186-188. Authors mentioned that "BIO treatment led to a dose-dependent increase of the overall cell number largely due to gain of "undifferentiated" LP cells." Puromorphamine treatment also led to a dose-dependent increase of the overall cell number (though not due to a gain of undifferentiated cells, but rather LP cells), yet the assessment of puromorphamine was described only as "had the opposite effect" of cyclopamine, on Line 184-185.

>> We omitted the sentence: “Treatment with purmorphamine had the opposite effect” in the revised version of the manuscript. Instead, we now write: “Treatment with purmorphamine led to a dose-dependent increase of both differentiated SMCs and LP fibrocytes” and then: “BIO treatment led to a dose-dependent increase of the overall cell number largely due to gain of “undifferentiated” LP cells”. <<

Figure 4.
* Line 213-215. "Purmorphamine led to a reciprocal dose-dependent increase of both SMCs and LP fibrocytes but SMCs were disproportionally increased." Consider indicating that the SMCs were disproportionately increased at 10uM.

>> We corrected this statement and now write: “Purmorphamine led to dose- dependent increase of both SMCs and LP fibrocytes; SMCs were disproportionally increased at 10 µM”.
<<

* Line 215-216. "Inhibition of WNT signaling by IWP-2 led to a dose-dependent equal reduction of LP fibrocytes and SMCs." SMCs; however, do not appear to be affected by IWP-2 treatment.

>> We provide a corrected statement on the effect of IWP-2 treatment which reads: “Inhibition of WNT signaling by IWP-2 led to a dose-dependent reduction of LP fibrocytes; SMC number reduction was significant at 5 and 10 µM (right hand panel)”.
<<

* Line 221-223. BMP4 treatment significantly impacts LP and SMC ratio at the two highest doses, but is not mentioned in the summary of findings.

>> The effect of BMP4 treatment on the ratio of SMCs to LP cells is significant but still rather weak and probably due to a slight but insignificant increase of total SMC number. Moreover, a clear dose-response curve is missing. We now write: “Inhibition of BMP4 signaling led to a slight increase of ALDH1A2$^{+}$ LP fibrocytes (peaking at 5 µg/ml NOG). BMP4 treatment increased the ratio of SMCs to LP fibrocytes at 100 and 250 ng/ml, probably to a slight (but insignificant) increase of the total SMC number. In either case, the effect was weak and lacked a clear dose- response curve.”
And in the summary we write: “BMP4 signaling seems to slightly favor SMCs over LP fibrocytes, while RA signaling is irrelevant for postnatal LP development.“ <<

Instead, WNT signaling is mentioned even though WNT signaling does not affect the ratio of LP and SMCs at any dose. Furthermore, it would be simple to state that these findings suggest that SHH signaling is required for maintenance of ALDH1A2+ LP cells (rather than state "These

© 2025. Published by The Company of Biologists under the terms of the Creative Commons Attribution License (https://creativecommons.org/licenses/by/4.0/).

findings suggest that SHH signaling is required for ALDH1A2 expression, hence differentiation of LP fibrocytes," because this was a maintenance stage experiment.

>> To accommodate the concern of the reviewer we now write in the summary or this section:

“These findings suggest that SHH signaling alone is required for maintenance of $ALDH1A2^{+}$ LP fibrocytes, whereas both SHH and WNT signaling account for the increase of the number of LP fibrocytes after birth.” <<

Figure 5.

* "Combined treatment with cyclopamine and IWP-2 led to an almost complete loss of ALDH1A2+ LP fibrocytes and a strong reduction of ACTA2+ SMCs, enhancing the effects of individual pathway inhibition." It is difficult to appreciate how combined treatment ENHANCED the effects of individual pathway inhibition given that cyclopamine treatment alone at 5uM led to an average of 0 LP cells, and at 10uM led to an average of 1 LP cell (Fig 4A, Table S9).

>> The reviewer is right. The concentrations chosen for cyclopamine and IWP-2 in this experiment were too high to make a statement that the two pathways enhance each other.

We have redone this experiment and choose a concentration for cyclopamine (1 µM) which did not lead to an effect in individual inhibition (Fig. 4A) and IWP2 (1 µM) which led to a weak reduction of LP fibrocytes. Co-administration of both compounds led to a reduction of both LP fibrocytes and SMCs supporting the notion that the pathways cooperate in LP and SMC expansion in PO ureters. This is shown in new Fig. 5A-C, Table S18. <<

* Consider re-ordering the data in Fig. 5 to align with how it is introduced in the main text (ie. 1) Control, 2) IWP-2/Cyclo, 3) BIO/Puro, 4) IWP-2/Puro, 5) Cyclo/BIO).

>> We have reordered the data as suggested by the reviewer. <<

* There appears to be a typo in labeled concentration of Puromorph. in Fig 5. panel A (10uM v 2uM). The image shows 10uM, but the graph reports 2uM. If 2uM was used, why was that concentration of puro chosen when 10uM, but not 2uM showed a significant increase in LP AND SMCs in Fig. 4?

>> We have redone these experiments using a concentration of 10 µM purmorphamine, and labeled the figures and graphs accordingly. <<

* Line. 235. "WNT signaling activation rescued LP cell number but not ALDH1A2 expression when SHH signaling was inhibited." Consider revising: WNT signaling activation did not rescue ALDH1A2+ LP cell number when SHH signaling was inhibited, but did expand the number of undifferentiated LP cells.

>> We have revised the sentence as suggested by the reviewer. <<

* Line 237-238. "We conclude that SHH and WNT signaling act in parallel in postnatal LP development." In my opinion, the data suggest that WNT promotes expansion of undifferentiated LP cells, while SHH promotes LP differentiation.

>> We agree with the reviewer and have accordingly changed our wording to “ We conclude that in early postnatal ureter development SHH signaling accounts for differentiation of LP cells, whereas SHH signaling cooperates with WNT signaling in expansion of LP cells and SMCs.” <<

* Line 225-226. A little confusing to title this results section "LP development," when previously using E12.5 explants for LP development and P0 explants for LP maintenance studies. Would E12.5 explants be better suited for the SHH/WNT study?

© 2025. Published by The Company of Biologists under the terms of the Creative Commons Attribution License (https://creativecommons.org/licenses/by/4.0/).

>>Analyzing P0 ureters addresses postnatal LP development. We have therefore revised the title of the section relating to Fig.4 into “SHH and WNT signaling are required for postnatal LP development“, and rephrased a couple of sentences to better describe this fact.
LP expansion occurs mainly after birth. Hence, E12.5 ureters would not be suited to study the combined affect of SHH and WNT signaling for this phase of development.<<

* In my opinion, pseudocolor the IF markers: ACTA2 (green), and ALDH1A2 (red) to match the 1) bar colors for the graphs in Fig. 5 panel E, 2) images in Fig. 5 panel A, and 3) Figures S4 and S5.

>> Good point, thank you. We have changed the color code as suggested by the reviewer.
<<

* Line 243-244. "In E14.5 and E18.5 explants, normal patterning into LP cells and SMCs occurred." There graphs show statistically significant changes in ALDH1A2+ LP cells and ACTA2+ SMCs, which makes it difficult to appreciate normal patterning occurred.

>> Please note that we redid the experiments with NOG/purmorphamine co- treatment shown in Fig. 5D,E with E12.5+2 days and P0 ureter explants to stay in line with the stages used in the previous experiments.
We omitted the statement on normal patterning, and merely describe the effect on ACTA2/ALDH1A2 expression and the changes in the number of LP and SMCs in the revised version of the text.
This now reads: „In E12.5 ureteral explants co-treated with purmorphamine (10 µM) and NOG (10 µg/ml), all mesenchymal cells exclusively expressed ALDH1A2 after 6 days of culture. In E12.5+2d explants, the number of ALDH1A2$^+$ LP fibrocytes and ACTA2$^+$ SMCs was increased; some mesenchymal cells coexpressed both markers.“<<

Furthermore, the expression of ACTA2 is reduced (would require WB or qPCR to assess expression levels) and the SMCs appear disorganized, which suggests BMP4 is important for 'maintaining' the SMC phenotype.

>> We do not agree with the statement of the reviewer that ACTA2 expression is reduced and that SMCs appear disorganized. At the level of resolution chosen for this experiment, we do not see an effect on the SMC phenotype. We therefore conclude:
„This suggests that BMP4 suppresses SHH-induced ALDH1A2 expression in uncommitted mesenchymal progenitors at E12.5. In committed progenitors and even more in differentiated SMCs (P0), BMP4 signaling does not seem to be required to maintain the SMC phenotype, nor does it have a major repressive effect on ALDH1A2 expression and LP fate.“ <<

* "Ectopic SHH signaling and ALDH1A2 expression cannot overwrite the SMC phenotype." Awkward summary statement considering this experiment did not simply address ectopic SHH signaling.

>> We omitted this sentence from the summary.<<

Figure 6.
* Line 254. Why were E18.5 explants used for this study which is aimed at addressing "the observed changes in cell numbers" and why were signaling inhibitors used for 48h for this study?

>> We redid the entire experiment with P0 ureter explant cultures for 18 h to stay in line with the previous experiments.<<

Since the entire panel of inhibitors is used and the most prominent changes in cell number occurred in Fig. 3, this experiment would make more sense if modeled in the same way and the data would flow better if presented immediately following that figure. E12.5 LP is as difficult to discern as the E18.5 LP.

>> The effect of all of this pathway inhibitors on proliferation in the undifferentiated UM

© 2025. Published by The Company of Biologists under the terms of the Creative Commons Attribution License (https://creativecommons.org/licenses/by/4.0/). 

was studied before (Bohnenpoll et al., 2017a, Mampo et al. 2017, Bohnenpoll et al., 2017b; Trowe et al., 2012) while the effect on postnatal expansion of the LP was not known. Moreover, the strongest (proliferative!!) expansion of the LP occurs between P0 and P7, i.e. exactly at the stages we wish to study here.
It is therefore very reasonable if not mandatory to study the effect of the pathway inhibitors on LP proliferation at P0, and this flows well after the analysis of pathways in Figure 4 and Figure 5. <<

* I can't find where the definition of the annotation is panel A is defined, but the elliptical annotation is not an authentic representation of the basement membrane. However, this type of annotation that demarcates the space between the TM and UE basement membrane would be helpful, here and elsewhere.

>> We have completely redone this analysis with P0 ureters as mentioned above. To address the concern of the reviewer that the ureteric epithelium should be better demarcated, we have performed an ACTA2-CDH1 co-staining to label SMCs and the epithelium (Fig. 6A,B). <<

Figure 7.
* Line 266. Why change IWP-2 to 10uM when 5uM has been used previously in E18 explant?

>> To stay coherent in our assays, we have redone the transcriptional profiling with P0 ureters treated with 10 µM cyclopamine and 5 µM IWP-2. <<

* Line 275. Clarify 104 downregulated DEGs.
>> We now write: „Using the same filtering conditions, 65 genes with reduced expression were found in IWP-2 treated ureters“.<<

* Unclear why the microarray is E18.5 + 18h treatment, while in situ hybridization validation is E14.5, P0, and P0 + 18h explants.
>> To stay coherent in our assays, we have redone the transcriptional profiling with P0 ureters treated with 10 µM cyclopamine and 5 µM IWP-2.
We included P0 ureters to have in vivo expression as a reference. We deleted the E14.5 stainings to stay coherent. <<

* Consider changing the format of Fig. 3 and Fig. S4, and Fig. 4 and Fig. S5. It would be helpful to include the left side image panels from Fig. S4 next to the left side graphs from Fig. 3 for the E12.5 explants. Likewise, it would be helpful to include the left side image panels from Fig. S5 next to the left side graphs from Fig 4 for the P0 explants. The main text makes little mention of the CDH1 ureteral urothelium data, so the Figs. 3/4 right side graphs may fit better in the data supplement with the images pulled from the right sides of Fig. S4/S5.

>> We have reorganized Figure 3 and 4 and the corresponding supplemental figures (Fig. S6, Fig. S7) as suggested by the reviewer. <<

* Figure legends report "two-tailed Student's t-test, Welch's t-test or Mann-Whitney U test" - which statistical test is indicated on the graphs?

>> We explain in the method section how the statistical analysis was performed. The supplemental tables contain the information on the specific statistical test used for each experiment. <<

Discussion
* Comments outlined for each Figure point to criticisms that limit the impact of some of the points made by the authors in the discussion. Overall, the discussion section of this manuscript is not nearly as exhaustive as the data presented in this manuscript. A careful discussion of the data presented in this manuscript would require more attention to 1) development vs maintenance phases examined, 2) attention to the order in which topics are presented, and 3) a careful comparison of the findings and those in the literature, including important technical details and contexts.

© 2025. Published by The Company of Biologists under the terms of the Creative Commons Attribution License (https://creativecommons.org/licenses/by/4.0/).

>> We have completely rewritten the discussion and expanded it to better cover the topics addressed in the experiments, particularly the early fetal phase of LP development (differentiation and diversification of the common progenitor) and the later postnatal expansion phase. We do not exactly know what the reviewer means with „careful comparison of the findings and those in the literature, including important technical details and contexts“ but we hope that we have covered the relevant, yet sparse literature. <<

* Line 301. LP is referred to as developing into a "thick cell layer" at early postnatal stages. In my opinion, the ureteral LP does not appear thick at either P0 or P7 - perhaps thickness measurements would support the opportunity to say there is a statistically significant increase in thickness. Otherwise, I suggest dropping the word thick, and referring to the emergence of a morphologically distinct LP.

>> We have performed measurements of the number of cells in the LP and the thickness of this cell layer (see new Figure 1E,F). This measurements clearly show an increased thickness of the LP from P0 to P7 and from P7 to P40. <<
* Claims that inhibition of SHH and WNT signaling abolished the LP do not seem appropriate when undifferentiated LP cells persist. Instead, inhibition of SHH and WNT signaling led to a dramatic loss of ALDH1A2, which represents a state of differentiation for the LP cells.

>> We have changed our discussion and have avoided this sentence. <<

* Line 329-335. This is a cumbersome sentence.
* Line 326. Should this say: In explants of E12.5/P0 ureters?

>> We have rewritten the discussion and omitted these sentences. <<

* Final sentence of the discussion ("…contribute to fibrotic programs that occur in the ureter during injury or obstruction") seems random without a little more effort to integrate this into the discussion.

>> We have rewritten the discussion and omitted this sentence. <<

Methods.
* Please mention the software and methods used to quantitate positive and negative cell counts. This is important considering that with the images presented, the boundaries of the cell types (especially when the manipulation impacts the LP and UE).

>> We have largely extended the paragraph on „Quantification and statistical analysis“ in the method section to more carefully explain how the quantifications were done. <<

* Define the conditions for the 'controls' for the combination experiments (Ethanol/DMSO/W etc.).

>> We had listed the solvents for all used compounds in the method sections but now additionally write: „Medium containing the respective solvents (control) or compounds was refreshed every second day.“

---

<u>Second decision letter</u>

MS ID#: dev.204214R1

MS TITLE: Interplay of SHH, WNT and BMP4 signaling regulates the development of the lamina propria in the murine ureter

AUTHORS: Philipp Straube, Anja Beckers, Ulrich Jany, Florian Bergmann, Timo Lüdtke, Carsten Rudat, Mark-Oliver Trowe, Imke Peters, Maximilian G. Klopf, Tamrat Mamo and Andreas Kispert

© 2025. Published by The Company of Biologists under the terms of the Creative Commons Attribution License (https://creativecommons.org/licenses/by/4.0/). 

I am happy to tell you that your manuscript has been accepted for publication in Development, pending our standard publication integrity checks.

Reviewer 1

*Advance summary and potential significance to field*

In the revised manuscript, the authors have conducted several new experiments whose results robustly reinforce their initial findings and offer additional support for their conclusions regarding the molecular regulation of lamina propria development and maintenance in the ureter wall. Key experiments using ureteric organ cultures have been re-performed using compound concentrations that are consistent across experiments. The Discussion section has been carefully edited, with refined interpretations of the roles of SHH and WNT signaling in lamina propria differentiation and proliferation. Additionally, the Methods section has been comprehensively updated to provide a detailed description of the statistical methods employed in the study.

Overall, the inclusion of new experimental data, combined with the thoughtful revisions to the text, has substantially enhanced the quality and rigor of their original work.

*Comments for the author*

The authors responded to this reviewer's comments. I have no further concerns beyond the first revision.

Reviewer 2: The authors have addressed or appropriately defended all concerns raised during the initial review of this manuscript in a satisfactory manner.

minor: Add a color legend for the antibodies used in Fig S7.

© 2025. Published by The Company of Biologists under the terms of the Creative Commons Attribution License (https://creativecommons.org/licenses/by/4.0/).
